# Characterising nutritional composition and labelling of packaged infant foods in Canada

**DOI:** 10.1017/jns.2025.10037

**Published:** 2025-09-11

**Authors:** Luiz Fernando Ceccon, Maryam Kebbe

**Affiliations:** 1 Department of Health Sciences, Federal University of Paraná, Curitiba, Paraná, Brazil; 2 Faculty of Kinesiology, University of New Brunswick, Fredericton, New Brunswick, Canada

**Keywords:** Cross-sectional studies, Food labelling, Food quality, Infant, Infant food, Nutrients, Public health

## Abstract

This cross-sectional study evaluated the nutritional composition and labelling of commercial foods in Canada targeted to infants up to 18 months of age. Front-of-package labelling requirements were assessed based on daily values identified by Health Canada for saturated fatty acids, sugars, and sodium for children aged one year and older. Infant commercial food products were identified from online and in-person records of retailers across Canada. A total of 1,010 products were identified. Products aimed at older infants (12–18 months) contained significantly more calories, macronutrients, sugars, saturated fat, and trans fat compared to those targeted at younger infants (<12 months). In addition, 40% of products for children aged 12–18 months required a ‘high in sugar’ front-of-package label, while less required a ‘high in saturated fats’ (13%) and ‘high in sodium’ (5%) label. Organic products had higher added sugar and fibre, while they were lower in calories, total fat, saturated fat, and protein. Plant-based products, including vegetarian/vegan products, contained fewer calories, fat, saturated fat, trans fat, and protein, but more fibre. Gluten-containing products had more calories, macronutrients, sugar, fibre, and saturated fat. Non-GMO labelled products had more calories, carbohydrates, and sugar, but less saturated fat. Significant differences were observed for vitamins and minerals across food categories (*p* < 0.05). Our findings offer valuable guidance for parents, caregivers, and healthcare professionals on infant nutrition, highlighting the importance of selecting foods that align with infants’ specific dietary needs.

## Introduction

Early childhood, particularly the first two years of life, is a critical period for brain development, language acquisition, and sensory pathways. During this stage of life, the timing and composition of nutrient intake are important. Undernutrition and overnutrition alike can cause health issues such as type 2 diabetes, hypertension, asthma, and obesity, and incur long-term socioeconomic impacts^(1)^.

The Canadian Nutrition for Healthy Term Infants statement highlights the importance of feeding human milk exclusively for the first six months of life, complemented with solid food from six months up to two years of age for optimal nutrition, immunologic protection, growth, and development^(2)^. Key recommendations include gradually increasing the frequency of complementary foods, beginning with iron-rich options, and introducing varied and lumpy textures by nine months of age^(2)^. The period of complementary feeding is a vital developmental stage that shapes a child’s long-term dietary habits, as evidenced by previous studies linking early fruit and vegetable introduction to increased consumption of these foods later in childhood^(3,4)^.

Commercially prepared infant foods are popular worldwide for their convenience and minimal preparation^(5)^. These mass-produced products, marketed as ‘convenient’ and ‘easy to feed’, often lead parents to make quick purchasing decisions, without thoroughly considering the nutritional content, quality, or long-term impact on their child’s diet and health^(6)^. Concerns have been raised about these products’ nutrient composition, labelling, and marketing practices^(7)^, including higher levels of added sugar and sodium than homemade foods, and misleading marketing regarding their actual nutritional content^(7,8)^. Additionally, the past decade has seen a surge in the consumption of plant-based, vegan, vegetarian, gluten-free and non-genetically modified organisms (GMO) labelled products, a trend that has extended to the infant feeding market^(9–11)^. Considering this growth, it is crucial to ensure that these products meet the specific dietary needs of developing children before constituting a major part of their diet.

There is a need for a thorough evaluation of the nutritional profiles of commercial infant foods in Canada. This research provides a comprehensive assessment of nutritional content and labelling of commercial foods targeted to infants up to 18 months of age. It examines nutritional differences across various categories, including age-specific ranges, organic, plant-based, vegan and vegetarian, gluten-free, and non-GMO products, along with an analysis of front-of-package (FOP) labelling requirements. This study aims to enhance our understanding of how various types of infant foods meet nutritional needs, supporting future updates to infant nutrition formulations and recommendations.

## Methods

### Study design

A cross-sectional study was conducted in May 2024 to assess the nutritional composition of solid foods and beverages targeted to infants up to 18 months of age in Canada. Eighteen months was chosen as the cut-off given the limited number of products advertised to infants beyond this age.

### Data collection and operationalisation

A comprehensive web search was conducted to identify a wide range of Canadian retailers—supermarkets, superstores, and drugstores—that offered infant-specific food products. The search utilised commonly used search engines (e.g. Google) and was guided by keywords such as ‘baby food’, ‘infant food’, ‘toddler meals’, and ‘infant cereal’ combined with ‘Canada’ and specific province names to ensure broad geographic coverage. To supplement the web search, common Canadian retailers were also identified through team member input, leveraging their familiarity with well-known national and regional store chains. This approach ensured inclusion of both major retail platforms and regionally relevant outlets.

All identified online retail platforms were systematically searched to capture all available infant food products that met inclusion criteria. In addition, in-person screenings were conducted in all major retail stores in Fredericton, New Brunswick, to identify products that may not have been available through online listings. In-person data collection followed a standardised protocol, which involved photographing product packaging—including Nutrition Facts tables and ingredient lists—and recording relevant product details.

All products identified were systematically reviewed to extract detailed labelling and nutritional information. Data were then recorded and organised using a structured digital data management platform, including product name, brand, weight, product category (e.g. baby cereals), food category (e.g. organic), nutrition facts, and nutrition labels.

Nutritional information was collected for serving size, caloric content, macronutrients, micronutrients, and sterols (cholesterol). Macronutrients included total fat (saturated fats, trans fats, monounsaturated fatty acids, polyunsaturated fatty acids), carbohydrates (sugars, added sugars, fibre), and protein. Although added sugars are not currently part of Canada’s mandatory labelling requirements, some products included this information in the Nutrition Facts table—likely because they were manufactured in the United States, where such labelling is required, and the same formulation was distributed in Canada. To comprehensively assess the nutritional landscape of commercial infant foods available to Canadian consumers, all products accessible for purchase in Canada were included in the analysis, irrespective of their country of manufacture. Minerals were categorised into macrominerals (calcium, magnesium, phosphorus, potassium, and sodium) and microminerals (choline, copper, iron, iodide, manganese, selenium, and zinc). Vitamins included vitamin A, B1, B2, B3, B5, B6, B7, B9, B12, C, D, E, and K. For products that indicated only the percentage of daily values (DV) or the amount per serving size for vitamins and minerals, the absolute gram amount was calculated proportionally based on the DV provided by Health Canada’s Technical Documents on Nutrition Labelling^(12)^.

Product categorisation followed industry-defined classifications commonly used in marketing commercial infant foods—namely baby cereals, baby food pouches and jars, baby snacks, toddler foods, and yogurt or similar dairy-based products. These product categories broadly aligned with the food groupings in the 2019 Canada’s Food Guide: baby cereals and snacks generally fell under whole grain foods; pouches and jars often contained vegetables, fruits, or protein foods; yogurt and similar products were considered protein foods; and many toddler foods included components from multiple categories, often emphasising protein.

Initial food categorisation was based on manufacturers’ package labelling and marketing descriptions—namely age group, organic, plant-based, vegetarian/vegan, gluten-free, non-GMO. To ensure accuracy, all classifications were verified through ingredient list review. For example, products without explicit plant-based or vegetarian/vegan labels were assessed by examining ingredients to determine appropriate classification. Similarly, gluten versus gluten-free status was assigned based on both manufacturer labelling and ingredient list inspection to confirm the presence or absence of gluten-containing ingredients. This dual approach allowed for accurate categorisation beyond reliance on labelling claims alone.

The following operational definitions were applied. Products were classified as *organic* if they met the criteria set by a recognised certification body (e.g. Canada Organic, USDA Organic). Organic foods are those produced without the use of synthetic pesticides, fertilizers, GMOs, antibiotics, or growth hormones. Organic certification typically ensures that both plant and animal ingredients meet these standards and that at least 95% of the product’s contents are organically produced^(13)^. *Plant-based* products are those whose primary ingredients originate from plants (e.g. grains, legumes, fruits, vegetables, nuts, and seeds), and which do not contain meat, poultry, or seafood. *Vegetarian* products exclude meat and fish but may include other animal-derived ingredients such as dairy or eggs. *Vegan* products exclude all animal-derived ingredients, including dairy, eggs, honey, and gelatin. In this study, due to substantial overlap in labelling and ingredient profiles, products labelled as vegetarian or vegan were grouped under a single category and confirmed by the absence of animal-sourced ingredients in the ingredient list. Products labelled as *gluten-free* are those that do not contain gluten, a group of proteins found in wheat, barley, rye, and their derivatives. Health Canada requires that foods labelled ‘gluten-free’ must not contain any gluten protein, modified gluten protein, or gluten-contaminated ingredients, and must have less than 20 parts per million (ppm) of gluten^(14)^. *Non-GMO* (non-genetically modified organism) products are those that do not contain ingredients derived from crops or organisms whose genetic material has been altered through genetic engineering techniques. Verification may come from programmes such as the ‘Non-GMO Project Verified’ seal, which ensures that a product meets specific criteria for GMO avoidance.

Statements and claims were categorised based on Canadian Food Inspection Agency guidelines for health claims on food labels, including those related to nutrient content, nutrient function, disease risk reduction, probiotics, and prebiotics^(15)^. Natural labels included statements such as ‘natural ingredients’, ‘no artificial ingredients’, ‘no artificial colors’, ‘no artificial preservatives’, and ‘all natural’. Nutrient content statements and claims describe the level of a nutrient or energy in foods, while nutrient function statements and claims highlight the recognised roles of specific nutrients in maintaining good health and supporting normal growth and development. Disease risk reduction claims are statements that link a food to a reduced risk of developing a diet-related disease or condition within the context of the total diet. According to Health Canada, prebiotics are non-viable food components that confer a health benefit on the host by modulating the microbiota, while probiotics are live microorganisms that, when administered in adequate amounts, confer a health benefit on the host^(15)^.

### Nutrient analysis

We performed comparative analyses by age group: under 12 months and 12 months and older. Health Canada categorises dietary reference intakes by age into three groups: 0–5 months, 6–11 months, and 12–36 months^(12)^. For infants under six months, dietary reference intakes should be met through human or formula milk, not solid foods. Although solid foods are typically recommended for introduction at six months^(2)^, this study includes products for infants as young as four months due to their limited market availability; only 14 products specifically targeted this age group. In addition, some products did not indicate a specific target age; rather, they used a range or descriptive terms to describe the infant developmental stage. Accordingly, these products were categorised into: (i) <12 months: Age ranges 4 months +, 6 months +, 7 months +, 8 months +, 9 months +, 10 months +, ‘beginners’, ‘crawlers’, ‘self-feeders’, ‘sitters’, ‘starter cereal’, and ‘supported sitter’ and (ii) ≥12 months: Age ranges 12 months +, 15 months +, and 18 months +. Products labelled as ‘for all ages’ or those lacking a clearly specified age range were excluded from analyses comparing products by age group (<12 months vs. ≥12 months; see Supplementary Table 1), given the potential for overlap across age categories. Similarly, products with labelling based on developmental milestones rather than discrete chronological ages were excluded from age-specific analyses due to the subjective nature of such indicators, which may vary across individuals and contribute to misclassification. However, these products were retained in analyses not stratified by age (e.g. organic, plant-based, vegan/vegetarian, gluten-free, and non-GMO), thereby maintaining a consistent total sample size across these comparisons.

We assessed the number of products requiring FOP nutrition labelling for sodium, total sugars, and saturated fat, focusing on prepackaged foods intended for children aged 1 to less than 4 years, per Health Canada’s Food and Drug Regulations^(16)^. Foods intended solely for infants aged 6 months to less than 1 year are exempt from FOP labelling, consistent with the absence of DVs for this age group and restrictions on including percentage DVs for macronutrients in Nutrition Facts tables. For products targeting children aged 1–4 years, FOP labelling is required when nutrient levels meet or exceed thresholds based on the reference amount or serving size (whichever is greater). For small reference amount foods (≤30 g or 30 mL, common in baby snacks or pouches), thresholds for FOP labelling are ≥1.0 g saturated fat (10% DV), ≥5.0 g total sugars (10% DV), or ≥120 mg sodium (10% DV). For general prepackaged foods (reference amount or serving size >30 g or 30 mL but <170 g or 170 mL), thresholds for FOP labelling are ≥1.5 g saturated fat (15% DV of 10 g), ≥7.5 g total sugars (15% DV of 50 g), or ≥180 mg sodium (15% DV of 1200 mg). For main dishes (≥170 g, applicable to some toddler foods), thresholds for FOP labelling are ≥3.0 g saturated fat (30% DV), ≥15.0 g total sugars (30% DV), or ≥360 mg sodium (30% DV). The Table of DVs specifies DVs for this age group: 10 g for saturated fat plus trans fat, 50 g for total sugars, and 1200 mg for sodium^(12)^. Products exceeding FOP labelling thresholds must display a ‘high in’ symbol for the respective nutrient(s).

### Statistical analysis

Descriptive statistics were performed to summarise the data, reporting measures of central tendency (mean) and dispersion (standard deviation). Independent samples t-tests were conducted using R 4.1.1 to evaluate differences in nutrient content across food categories. In-text results are reported only for comparisons in which the combined number of products across subgroups (e.g. organic and non-organic) represented at least 5% of the total sample. This criterion was applied consistently across all subgroup analyses. Analyses were conducted using nutrient values standardised to 100 g or 100 mL by converting reported nutrient contents per serving through proportional scaling (i.e. the nutrient amount was divided by the serving size in grams or millilitres and then multiplied by 100). Statistical significance was defined as *p* < 0.05.

## Results

### Infant food stores

A total of 80 stores were identified, including major supermarket chains, superstores, and drugstores. In Fredericton, New Brunswick, Canada, 10 stores had physical locations, of which 8 offered infant food options and were included in the study. Among the 70 stores without physical stores in Fredericton, 48 offered online shopping, with 42 offering infant food options. Thus, a total of 50 stores met our inclusion criteria. The geographical distribution of physical food stores selling infant food across Canadian provinces and territories is depicted in Supplementary Figure 1.

### Overall summary of products

A total of 1,010 products was identified. Table 1 provides an overall summary of the products. The average weight of the products was 107.92 ± 74.35 grams or 127.89 ± 52.17 millilitres. The largest product category was infant food pouches and jars, comprising 62.28% (*n* = 629) of total products.

**Table 1.** Overall summary of infant products

Categories	*n* (%)
*Type*	
Baby cereal	84 (8.32%)
Baby food pouches and jars	629 (62.28%)
Baby snacks	198 (19.60%)
Toddler foods	57 (5.64%)
Yogurt or similar	42 (4.16%)
*Age range*	
4 months +	14 (1.39%)
6 months +	352 (34.85%)
7 months +	51 (5.05)
8 months +	97 (9.60%)
9 months +	31 (3.07%)
10 months +	9 (0.89%)
12 months +	127 (12.57%)
15 months +	4 (0.40%)
18 months +	4 (0.40%)
All ages	8 (0.80%)
Subjective milestones	110 (10.89%)
Not mentioned	203 (20.10%)
*Organic category*	
Organic	728 (72.08%)
Non-organic	282 (27.92%)
*Plant-based category*	
Plant-based	750 (74.26%)
Non-plant-based	260 (25.74%)
*Vegetarian/vegan category*	
Vegetarian/vegan	747 (73.96%)
Not vegetarian/vegan	263 (26.04%)
Vegetarian/vegan label Labelled Unlabelled	127 (12.57%) 883 (87.43%)
*Gluten status*	
Gluten-free	800 (79.21%)
Gluten-containing	210 (20.79%)
*Gluten-free label*	
Labelled	262 (25.94%)
Unlabelled	748 (74.06%)
*Non-GMO label*	
Labelled	439 (43.47%)
Unlabelled	571 (56.53%)
Natural label	
Labelled	523 (51.78%)
Unlabelled	487 (48.22%)
*Nutrient content statements and claims*	
At least one	677 (67.03%)
Protein	85 (8.42%)
Fat, fatty acids and cholesterol	28 (2.77%)
Fibre	58 (5.74%)
Sodium/salt	247 (24.46%)
Sugars	455 (45.05%)
Other nutrients	254 (25.15%)
None	333 (32.97%)
*Nutrient function statements and claims*	
At least one	123 (12.18%)
Protein	8 (0.79%)
Fat	2 (0.20%)
Omega-3	8 (0.79%)
Vitamin A	9 (0.89%)
Vitamin B6	7 (0.69%)
Vitamin B9	4 (0.40%)
Vitamin C	7 (0.69%)
Vitamin D	19 (1.88%)
Calcium	2 (0.20%)
Iron	60 (5.94%)
Zinc	16 (1.58%)
Other nutrients	47 (4.65%)
None	887 (87.72%)
*Disease risk reduction claims*	
At least one	1 (0.10%)
None	1,009 (99.90%)
*Probiotic and prebiotic claims*	
Contains probiotic claims	25 (2.48%)
Contains prebiotic claims	9 (0.89%)
None	976 (96.63%)

A third of products were marketed as 6 months + (34.85%; *n* = 352). Interestingly, 14 products (1.39%) were labelled for infants as young as four months, which conflicts with Health Canada’s guidelines on complementary feeding^(2)^. According to these guidelines, while it is acceptable for labels to specify an age range for which a product is suitable, it is not permissible to indicate that infant foods are appropriate for infants under six months, except for infant formula and human milk fortifiers^(15)^. Additionally, 20.10% (*n* = 203) of products did not specify an age range and 10.89% (*n* = 110) of products indicated subjective milestones in place of age, such as crawlers, self-feeders, and sitters.

The majority of food products (72.08%; *n* = 728) were labelled as organic. Plant-based products accounted for 74.26% (*n* = 750) of the market, while vegetarian/vegan products represented 73.96% (*n* = 747), demonstrating an important overlap between these categories. Notably, only 12.57% (*n* = 127) of products had a vegetarian/vegan label, despite a large proportion of the products fitting these categories based on ingredients alone—likely reflecting manufacturer caution, certification requirements, or marketing choices. Nearly a quarter of products (25.94%, *n* = 262) had a gluten-free label. However, based on the listed ingredients alone, and assuming no risk of cross-contamination, most products (79.21%; *n* = 800) could be considered gluten-free, though traces from manufacturing cannot be ruled out. Products lacking non-GMO labelling were common (56.53%; *n* = 571).

In terms of natural labels, 51.78% (*n* = 523) of products contained a natural label. Approximately 67.03% (*n* = 677) of products contained nutrient content statements and claims. Among these, sugar claims were the most frequently mentioned (45.05%; *n* = 455). Protein claims were included for 8.42% (*n* = 85) of products. Claims for fats, fatty acids, and cholesterol were mentioned less frequently, appearing in only 2.77% (*n* = 28) of products. On the other hand, claims related to other nutrients, such as vitamins and minerals, appeared on 25.15% (*n* = 254) of products. Most products did not feature any nutrient function claims (87.72%, *n* = 887) and did not contain any probiotic or prebiotic claims (96.63%, *n* = 976). Disease risk reduction claims were exceedingly rare, with only 0.10% (*n* = 1) of products making the following statement: ‘Various studies suggest that blueberries have the potential to stave off cognitive decline and support memory function’.

### Overall summary of nutrition facts

Nutrition facts of included products are outlined in Table 2, with values standardised to 100 g or 100 mL, depending on whether the product’s nutrition facts were reported by weight or volume. Overall, 35 products contained added sugars, while 17 products explicitly stated ‘0 g added sugars’; the remaining products did not indicate the amount of added sugars on the nutrition facts table. In addition, less than 1% of products indicated levels of monounsaturated and polyunsaturated fatty acids. Cholesterol levels were low, averaging 12.48 ± 18.54 mg.

**Table 2.** Overall summary of nutrition facts standardised to 100 g or 100 mL

Nutrition facts	Amount †	% Daily value †
**Calories** (*n* = 1,010)	166.39 ± 157.50 kcal	–
**Macronutrients**		
Fat (*n* = 1,010)	2.94 ± 5.30 g	–
Saturated fat (*n* = 770)	0.56 ± 1.79 g	–
Monounsaturated fat (*n* = 3)	2.22 ± 1.86 g	–
Polyunsaturated fat (*n* = 6)	0.72 ± 0.37 g	–
Omega-3 (*n* = 3)	0.19 ± 0.08 g	–
Omega-6 (*n* = 1)	0.35 g	–
Trans fat (*n* = 730)	0.001 ± 0.01 g	–
Cholesterol (*n* = 84)	12.48 ± 18.54 mg	–
Carbohydrates (*n* = 1,010)	31.42 ± 29.12 g	–
Sugar (*n* = 1,008)	11.70 ± 12.42 g	–
Added sugar (*n* = 52)	10.95 ± 13.15 g	–
Fibre (*n* = 968)	2.73 ± 3.60 g	–
Protein (*n* = 1,010)	3.87 ± 4.79 g	–
**Macrominerals**		
Calcium (*n* = 930)	62.87 ± 129.19 mg	18.59 ± 44.90%
Magnesium (*n* = 75)	71.31 ± 102.86 mg	94.32 ± 137.07%
Phosphorus (*n* = 25)	307.12 ± 164.86 mg	108.48 ± 64.29%
Potassium (*n* = 910)	240.80 ± 213.43 mg	24.88 ± 25.22%
Sodium (*n* = 1,010)	43.15 ± 78.38 mg	–
**Microminerals**		
Choline (*n* = 41)	166.65 ± 175.47 mg	102.56 ± 109.04%
Copper (*n* = 27)	0.20 ± 0.24 mg	96.45 ± 111.17%
Iron (*n* = 934)	3.82 ± 9.42 mg	38.81 ± 95.18%
Iodide (*n* = 56)	25.72 ± 39.11 µg	1.91 ± 31.50%
Manganese (*n* = 33)	1.23 ± 3.28 mg	30.90 ± 51.35%
Selenium (*n* = 26)	10.74 ± 12.64 µg	49.47 ± 60.41%
Zinc (*n* = 113)	2.82 ± 3.15 mg	93.56 ± 101.84%
**Vitamins**		
Vitamin A (*n* = 181)	97.52 ± 312.97 µg	42.96 ± 62.93%
Vitamin B1 (*n* = 105)	1.06 ± 0.88 mg	311.59 ± 225.61%
Vitamin B2 (*n* = 77)	1.27 ± 0.77 mg	307.13 ± 186.32%
Vitamin B3 (*n* = 112)	10.47 ± 9.71 mg	234.97 ± 201.59%
Vitamin B5 (*n* = 21)	2.62 ± 2.11 mg	137.19 ± 106.75%
Vitamin B6 (*n* = 80)	0.39 ± 0.95 mg	85.42 ± 61.45%
Vitamin B7 (*n* = 45)	5.94 ± 6.06 µg	193.89 ± 100.30%
Vitamin B9 (*n* = 67)	46.69 ± 66.57 µg	70.26 ± 83.03%
Vitamin B12 (*n* = 74)	2.24 ± 15.45 µg	79.07 ± 84.54%
Vitamin C (*n* = 198)	22.76 ± 21.66 mg	54.82 ± 46.87%
Vitamin D (*n* = 101)	3.40 ± 3.89 µg	30.20 ± 38.71%
Vitamin E (*n* = 105)	4.31 ± 3.87 mg	79.22 ± 72.37%
Vitamin K (*n* = 23)	9.05 ± 12.77 µg	212.24 ± 389.67%

† Overall summary of nutritional facts standardised per 100 g or 100 mL serving. Values are expressed as mean ± standard deviation based on the values declared on product labels.

### Comparative analyses

#### Age: <12 months versus ≥12 months

Products targeted at infants aged 12 to 18 months contained significantly more calories (279.33 ± 160.72 kcal vs. 139.92 ± 142.52 kcal, *p* < 0.001), fat (6.78 ± 7.23 g vs. 2.26 ± 4.19 g, *p* < 0.001), saturated fat (1.12 ± 1.98 g vs. 0.33 ± 0.92 g, *p* < 0.001) and trans fat (0.01 ± 0.02 g vs. 0.00 g, *p* = 0.017) compared to products aimed at infants less than 12 months. Similarly, carbohydrates (50.60 ± 29.67 g vs. 26.69 ± 25.65 g, *p* < 0.001), sugars (17.52 ± 18.62 g vs. 10.22 ± 9.97 g, *p* < 0.001), and protein (6.03 ± 4.87 g vs. 3.39 ± 4.43 g, *p* < 0.001) were higher in the older age compared to the younger age group.

Among the 135 products targeted at infants aged 12 to 18 months, 54 (40.0%) would require a ‘high in sugar’ FOP label, 17 (12.6%) would require a ‘high in saturated fats’ FOP label, and 7 (5.2%) a ‘high in sodium’ FOP label.

Absolute amounts of vitamins and minerals had a varied distribution across age groups. Calcium (*p* = 0.010), potassium (*p* < 0.001), and sodium (*p* < 0.001) absolute amounts were significantly higher in the older age group, while magnesium (*p* = 0.017) and vitamin C (*p* < 0.001) were significantly lower, compared to the younger age group. Notably, sodium was four times higher in the older than the younger group (108.94 ± 131.46 mg vs. 27.41 ± 54.30 mg, *p* < 0.001). These differences were consistent when analysing %DV, with the same direction and significance, with three exceptions: %DVs for iron (*p* = 0.005) and vitamin B2 (*p* = 0.015) were significantly lower and higher, respectively, in products for younger compared to older infants, despite no significant differences in absolute amounts, whereas absolute levels of vitamin A (p = 0.001) were significantly higher in the younger compared to older group, despite no significant difference in %DV. A detailed comparative analysis by age is presented in Table 3 and Supplementary Table 1.

**Table 3.** Comparative analysis of products targeted for infants under 12 months and 12 months or older

Nutrition facts †	< 12 Months (*n* = 554)	≥ 12 Months (*n* = 135)
**Serving size** (g or mL)	100.00	100.00
**Calories** (kcal)	139.92 ± 142.52	279.33 ± 160.72*
**Macronutrients**		
Fat (g)	2.26 ± 4.19	6.78 ± 7.23*
Saturated fat (g)	0.33 ± 0.92	1.12 ± 1.98*
Monounsaturated fat (g)	3.29 ± 0.36	0.09
Polyunsaturated fat (g)	0.83 ± 0.31	0.09
Omega-3 (g)		0.19 ± 0.08
Omega-6 (g)		0.35
Trans fat (g)	0.00	0.01 ± 0.02*
Cholesterol (mg)	13.32 ± 13.55	9.10 ± 5.47
Carbohydrates (g)	26.69 ± 25.65	50.60 ± 29.67*
Sugar (g)	10.22 ± 9.97	17.52 ± 18.62*
Added sugar (g)	1.32 ± 2.26	16.94 ± 14.60**
Fibre (g)	2.51 ± 2.86	3.37 ± 4.89
Protein (g)	3.39 ± 4.43	6.03 ± 4.87*
**Macrominerals**		
Calcium (mg)	50.60 ± 122.92	87.31 ± 142.14*
Calcium (%)	18.67 ± 45.89*	12.48 ± 20.93
Magnesium (mg)	100.66 ± 135.32*	43.04 ± 23.28
Magnesium (%)	134.06 ± 180.64*	55.41 ± 31.81
Phosphorus (mg)	349.43 ± 173.47**	184.10 ± 55.16
Phosphorus (%)	126.67 ± 63.44**	31.53 ± 24.40
Potassium (mg)	223.73 ± 176.04	308.55 ± 253.32*
Potassium (%)	29.99 ± 23.88*	12.02 ± 9.40
Sodium (mg)	27.41 ± 54.30	108.94 ± 131.46*
**Microminerals**		
Choline (mg)	289.98 ± 208.29	
Choline (%)	184.75 ± 131.03	
Copper (mg)	0.24 ± 0.20	
Copper (%)	120.00 ± 103.32	
Iron (mg)	3.54 ± 8.96	4.97 ± 9.91
Iron (%)	33.20 ± 82.58	70.70 ± 142.07*
Iodide (µg)	28.39 ± 53.81	16.29 ± 2.74
Iodide (%)	22.01 ± 41.20	17.15 ± 3.91
Manganese (mg)	1.56 ± 1.91	1.01 ± 0.77
Manganese (%)	79.87 ± 79.30	76.38 ± 56.57
Selenium (µg)	2.02	14.29
Selenium (%)	10.10	71.43
Zinc (mg)	3.32 ± 3.69	1.91 ± 1.73
Zinc (%)	110.37 ± 122.06	65.35 ± 60.39
**Vitamins**		
Vitamin A (µg)	218.66 ± 381.12*	79.91 ± 89.42
Vitamin A (%)	45.91 ± 74.64	26.60 ± 30.47
Vitamin B1 (mg)	1.13 ± 0.61	1.60 ± 1.49
Vitamin B1 (%)	374.73 ± 202.68	316.01 ± 300.14
Vitamin B2 (mg)	1.56 ± 0.66	1.18 ± 0.93
Vitamin B2 (%)	392.04 ± 165.13*	236.69 ± 185.24
Vitamin B3 (mg)	12.97 ± 7.66	15.73 ± 15.57
Vitamin B3 (%)	325.86 ± 191.25	257.88 ± 263.04
Vitamin B5 (mg)	2.11 ± 1.38**	0.43
Vitamin B5 (%)	116.02 ± 77.03**	21.43
Vitamin B6 (mg)	0.55 ± 1.62	0.34 ± 0.20
Vitamin B6 (%)	82.96 ± 66.62	68.04 ± 39.15
Vitamin B7 (µg)	6.93 ± 6.99	6.32 ± 3.28
Vitamin B7 (%)	116.44 ± 117.16	79.44 ± 40.17
Vitamin B9 (µg)	31.33 ± 33.68	42.32 ± 60.74
Vitamin B9 (%)	67.27 ± 59.61	40.55 ± 61.86
Vitamin B12 (µg)	0.33 ± 0.42	13.69 ± 42.04
Vitamin B12 (%)	64.50 ± 83.11	51.62 ± 31.46
Vitamin C (mg)	24.44 ± 23.71*	11.68 ± 7.75
Vitamin C (%)	48.85 ± 47.21	78.26 ± 52.69*
Vitamin D (µg)	5.84 ± 5.22**	2.38 ± 2.61
Vitamin D (%)	58.41 ± 52.12**	14.29 ± 15.65
Vitamin E (mg)	4.34 ± 4.25	4.23 ± 2.70
Vitamin E (%)	84.51 ± 82.54	63.96 ± 44.13
Vitamin K (µg)	6.01 ± 10.62	23.50 ± 13.50
Vitamin K (%)	239.94 ± 424.94	80.70 ± 46.33

† Values are presented as mean ± standard deviation. Statistical significance is indicated as follows: * denotes *p* < 0.05; ** denotes *p* < 0.05 but with a sample size less than 5% of the total.

#### Organic versus non-organic

Organic products contained significantly fewer calories compared to non-organic products (155.98 ± 152.06 kcal vs. 193.26 ± 168.73 kcal, *p* = 0.001). In terms of macronutrients, organic products also had significantly less total fat (2.58 ± 5.05 g, *p* = 0.001), namely from saturated fats (0.48 ± 1.87 g, *p* = 0.043), compared to non-organic products (total fat: 3.87 ± 5.80 g; saturated fat: 0.74 ± 1.56 g). Protein content was also significantly lower in organic products than in non-organic products (3.49 ± 4.71 g vs. 4.86 ± 4.89 g, *p* < 0.001). However, organic products had significantly higher added sugar (12.97 ± 14.31 g vs. 4.87 ± 5.77 g, *p* = 0.001) and fibre (3.01 ± 3.59 g vs. 1.99 ± 3.52 g, *p* < 0.001) compared to non-organic products.

Significant differences in the absolute content of several vitamins and minerals were observed between organic and non-organic products. Organic products had significantly lower levels of calcium (*p* < 0.001), sodium (*p* < 0.001), iron (*p* < 0.001), and vitamin B3 (*p* = 0.015), while significantly higher levels were found for vitamin D (*p* < 0.001). These differences were consistent when analysing %DV, with the same direction and significance, with three exceptions: %DVs for vitamin A (*p* = 0.005) and vitamin B12 (*p* = 0.040) were significantly higher in organic products compared to non-organic products, whereas absolute levels of vitamin B9 (*p* = 0.012) were significantly higher in organic products compared to non-organic products, despite no significant difference in %DV. A detailed comparative analysis of organic versus non-organic products is presented in Supplementary Table 2.

#### Plant-based versus non-plant-based

Plant-based products had significantly fewer calories compared to non-plant-based products (155.72 ± 151.31 kcal vs. 197.17 ± 171.44 kcal, p < 0.001). They also contained significantly lower amounts of protein (2.96 ± 4.25 g vs. 6.49 ± 5.30 g, *p* < 0.001) and fat (2.11 ± 4.55 g vs. 5.32 ± 6.45 g, *p* < 0.001), including saturated fat (0.36 ± 1.76 g vs. 1.16 ± 1.73 g, *p* < 0.001), and trans fat (0.00 ± 0.00 g vs. 0.004 ± 0.02 g, *p* = 0.004) compared to non-plant-based products. Conversely, they had significantly higher fibre content (3.04 ± 3.85 g vs. 1.82 ± 2.56 g, *p* < 0.001). As expected, cholesterol was not reported in any plant-based products, while eight non-plant-based products had an average cholesterol content of 12.48 ± 18.54 g.

The absolute content of vitamins and minerals varied notably between the two groups. Plant-based products were significantly higher in zinc (*p* < 0.001), but lower in calcium (*p* < 0.001) and sodium (*p* < 0.001). In addition, plant-based products were significantly higher in vitamin D (*p* < 0.001), but lower in vitamins B2 (*p* < 0.001) and B3 (*p* = 0.0016), all compared to non-plant-based products. These differences were consistent when analysing %DV, with the same direction and significance, with three exceptions: %DVs for vitamins B6 (*p* = 0.014), B9 (*p* = 0.027), and B12 (*p* < 0.001) were significantly higher in plant-based compared to non-plant-based products, despite no significant difference in absolute values. A detailed comparative analysis of plant-based versus non-plant-based products is presented in Supplementary Table 3.

#### Vegetarian/vegan versus non-vegetarian/vegan

Vegetarian/vegan products contained significantly fewer calories compared to non-vegetarian products (155.70 ± 151.27 kcal vs. 196.76 ± 171.36 kcal, *p* < 0.001). Vegetarian/vegan products also contained significantly higher fibre compared to non-vegetarian/vegan products (3.04 ± 3.85 g vs. 1.85 ± 2.57 g, *p* < 0.001).

In terms of macronutrients, vegetarian/vegan infant products had significantly lower fat content (2.12 ± 4.56 g vs 5.27 ± 6.44 g, *p* < 0.001), including lower levels of saturated fat (0.36 ± 1.76 g vs. 1.15 ± 1.73 g, *p* < 0.001) and trans fat (0.00 ± 0.00 g vs. 0.004 ± 0.02 g, *p* = 0.003) compared to non-vegetarian/vegan products. Additionally, vegetarian/vegan products had significantly less protein compared to non-vegetarian/vegan products (2.96 ± 4.25 g vs. 6.46 ± 5.32 g, *p* < 0.001).

In terms of absolute content of vitamins and minerals, vegetarian/vegan products had significantly higher concentrations of zinc (*p* < 0.001), while they had significantly lower concentrations of calcium (*p* < 0.001) and sodium (*p* < 0.001). Vegetarian/vegan products contained significantly higher levels of vitamin D (*p* < 0.001). In contrast, they contained significantly less vitamins B2 (*p* < 0.001) and B3 (*p* < 0.001), all compared to non-vegetarian/vegan products. These differences were consistent when analysing %DV, with the same direction and significance, with three exceptions: %DVs for vitamins B6 (*p* = 0.014), B9 (*p* = 0.027), and B12 (*p* < 0.001) were significantly higher in vegetarian/vegan products compared to non-vegetarian/vegan products, despite no significant difference in absolute values. A detailed comparative analysis of vegetarian/vegan versus non-vegetarian/vegan products is presented in Supplementary Table 4.

#### Non-GMO labelled versus unlabelled

Products containing a non-GMO label had significantly more calories (184.19 ± 166.87 kcal vs. 152.71 ± 148.97 kcal, *p* = 0.002), carbohydrates (35.06 ± 29.89 g vs. 28.62 ± 28.22 g, *p* < 0.001), and sugar (13.19 ± 13.22 g vs. 10.56 ± 11.65 g, *p* = 0.001), but less saturated fat (0.40 ± 1.51 vs. 0.70 ± 1.99 g, *p* = 0.016) compared to unlabelled products.

Nearly all minerals present in at least 5% of products were found in significantly higher absolute amounts in the non-GMO labelled group: sodium (*p* = 0.015), iron (*p* < 0.001), and zinc (*p* = 0.015). The same trend was observed for absolute amounts of vitamins, with significantly higher values in non-GMO labelled products for vitamins B6 (*p* = 0.006) and B9 (*p* = 0.017) compared to unlabelled products. These differences were consistent when analysing %DV, with the same direction and significance, with two exceptions: %DVs for calcium (*p* = 0.023) and vitamin D (*p* = 0.017) were significantly higher in the non-GMO labelled products compared to the unlabelled group, despite no significant difference in absolute values. A detailed comparative analysis by GMO status is presented in Supplementary Table 5.

#### Gluten-free versus gluten-containing

Products containing gluten had significantly higher caloric content compared to gluten-free products (265.29 ± 171.49 kcal vs. 140.43 ± 143.06 kcal, *p* < 0.001). In terms of macronutrients, gluten-containing products had greater fat content (5.92 ± 6.13 g vs. 2.16 ± 4.76 g, *p* < 0.001) including saturated fat (1.18 ± 2.21 g vs. 0.43 ± 1.66 g, *p* < 0.001). Furthermore, gluten-containing products were significantly richer in carbohydrates (45.77 ± 28.89 g vs. 27.66 ± 28.00 g, *p* < 0.001), sugar (13.02 ± 9.81 g vs. 11.35 ± 13.00 g, *p* = 0.042), and fibre (3.45 ± 3.44 g vs. 2.54 ± 3.62 g, *p* < 0.001). They also contained significantly more protein (6.76 ± 5.34 g vs. 3.11 ± 4.34 g, *p* < 0.001), all compared to gluten-free products.

Absolute amounts of macrominerals were significantly more abundant in the gluten-containing than the gluten-free group, especially calcium (*p* < 0.001), magnesium (*p* = 0.011) and sodium (*p* < 0.001). Conversely, microminerals absolute amounts varied according to each group. Iron was significantly higher in the gluten-containing group (*p* < 0.001), while iodide was significantly lower in the gluten-containing group (*p* = 0.038), all compared to gluten-free products. Gluten-containing products also had higher absolute amounts of vitamins B2 (*p* < 0.001), B3 (*p* = 0.018), and D (*p* = 0.004) compared to gluten-free products. On the other hand, gluten-containing products had significantly lower levels of vitamin E compared to gluten-free products (*p* = 0.040). These differences were consistent when analysing %DV, with the same direction and significance, with one exception: %DV for vitamin B6 (*p* = 0.044) was significantly higher in the gluten-containing products compared to gluten-free products, despite no significant difference in absolute values. A detailed comparative analysis by GMO status is presented in Supplementary Table 6.

## Discussion

Our study aimed to evaluate and compare the nutritional composition of commercial infant food products across various categories, including age groups, organic vs. non-organic, plant-based vs. non-plant-based, vegetarian/vegan vs. non-vegetarian/vegan, non-GMO labelled vs. unlabelled, and gluten-free vs. gluten-containing products. Our findings offer critical insights into the nutritional landscape of these products and highlight important considerations for informing evidence-based recommendations and regulatory efforts in infant feeding in Canada.

Age group analyses revealed that products targeted at older infants contained significantly higher levels of energy, fats, carbohydrates, sugars, and protein compared to those marketed for younger infants. Products in the older age group more commonly featured mixed dishes such as pasta with meat, rice with vegetables, or textured entrées, while those intended for younger infants predominantly consisted of simple fruit or vegetable purées. These distinctions reflect the evolving dietary patterns over the first year of life and are consistent with Canadian infant feeding recommendations, which support the progressive introduction of iron-rich, protein-dense, and diverse foods and textures to meet infants’ increasing nutritional and developmental requirements^(2)^.

Interestingly, among products targeted at children aged 12 to 18 months, two-fifth would require a ‘high in sugar’ FOP label, raising concerns about early-life exposure to sweet tastes and potential long-term dietary habits. The proportion of products requiring FOP labels for saturated fat and sodium was notable, though lower, with 13% and 5% of products qualifying for ‘high in saturated fat’ and ‘high in sodium’ nutrition symbol, respectively. This prevalence of FOP labels—especially those related to sugar content—underscores ongoing nutritional challenges in commercial infant foods, reflecting similar concerns previously documented in adult food products^(17)^. As of January 1, 2026, Health Canada mandates that prepackaged foods meeting or exceeding specified thresholds for saturated fat, sugars, or sodium display a FOP nutrition symbol^(16)^. This symbol aims to help consumers quickly identify foods high in these nutrients, which are associated with increased health risks when consumed in excess.

Most infant food products were labelled as organic. Food manufacturers may be targeting a strong consumer preference for organic options in the baby food market, likely driven by perceptions of healthier status^(18)^. Compared to organic products, non-organic products were found to have higher levels of calories, total fat, saturated fat, and protein. These findings complement previous studies suggesting higher additives and processed ingredients in non-organic products to enhance flavour and shelf life^(19)^. While organic products were higher in sugar, this may reflect a greater reliance on fruit purées or concentrates to provide natural sweetness in place of additives or fortificants, rather than the addition of refined sugars. Additionally, organic products in our analysis contained higher levels of vitamins A, B9, B12, and D as well as fibre. These findings complement previous studies showing greater levels of certain nutrients in organic products, such as vitamin C, iron, magnesium, and phosphorous than non-organic varieties of the same foods^(20)^. Collectively, these findings support the notion that organic farming practices may enhance the nutrient profile of foods by promoting soil health and biodiversity^(21)^. These practices include the use of fewer pesticides, no GMOs, and adherence to stricter farming standards^(22)^. A systematic review suggests that organic intake is associated with reduced incidence of several conditions and diseases, such as infertility, birth defects, and pre-eclampsia^(23)^. Nonetheless, further longitudinal studies are required to assess the long-term health benefits of organic food consumption in infants and toddlers.

While organic products encompass broader standards, including restrictions on pesticides, fertilizers, and animal welfare, non-GMO refers only to the absence of genetically modified organisms^(22)^. Products with a non-GMO label contained more calories, carbohydrates, and sugars but less saturated fat compared to unlabelled products. The higher levels of certain vitamins and minerals in non-GMO labelled products may indicate manufacturers’ efforts to meet consumer expectations for higher nutritional quality, driven by the belief that non-GMO products are healthier than their GMO counterparts^(24)^. However, it is crucial to recognise that the non-GMO label alone does not guarantee superior nutritional quality, as other factors related to processing methods play significant roles^(25)^. Further research could help determine whether non-GMO labelling directly correlates with enhanced nutritional quality or if other factors contribute to these disparities.

In our study, plant-based products, vegetarian, and vegan products had lower caloric content, protein, and fat (including saturated and trans fats), but higher fibre compared to their counterparts. These results are in line with the broader literature indicating that plant-based diets tend to be lower in calories and fats while being richer in dietary fibre and essential micronutrients^(26)^. Nutrients of concern with diets that are more restrictive in animal-source foods include vitamin B12, vitamin D, iron, zinc, and iodine^(27)^. Interestingly, our analysis showed that both plant-based and vegetarian/vegan products—which largely overlapped—contained significantly higher levels of vitamins B6, B9, B12, and D, as well as zinc, but lower levels of vitamins B2 and B3, calcium, and sodium. Although the elevated levels of vitamins B12 and D may seem unexpected—given their common classification as nutrients of concern in restrictive diets—this likely reflects the widespread use of fortification strategies in commercial infant products to offset the absence of animal-source ingredients, particularly in products marketed as nutritionally complete alternatives. While fortifying infant products with nutrients is promising, it is important to consider overall daily nutrient intake to ensure dietary reference needs are met. Indeed, children consuming plant-based diets risked inadequate intakes of vitamin B12, iron, and zinc^(28)^. In adults, intake of vitamin B12, vitamin D, iron, zinc, iodine, and calcium were generally lower in plant-based compared to non-plant-based diets^(28)^, with specific concerns for bone health^(29)^. These results suggest a need for careful dietary planning to ensure adequate intake of these nutrients from other sources ^(30)^.

Gluten-containing products exhibited significantly higher caloric content, fats, carbohydrates, sugars, fibre, and protein compared to gluten-free products. This finding is consistent with the literature indicating that gluten-containing grains, such as wheat, are a rich source of these macronutrients^(31)^. The higher nutrient density observed in gluten-containing products, especially in baby cereals (71/84 gluten-containing), underscores their role in meeting the developmental needs of infants. A review of existing data shows that there are detrimental effects to following a gluten-free diet, including loss of dietary fibre, deficiencies in dietary minerals and vitamins, and potential heavy metal exposure^(32)^. Nevertheless, in certain populations, such as infants with celiac disease or gluten sensitivity, gluten-free products are essential and require careful fortification to ensure nutritional adequacy^(33)^.

Although we observed differences in macronutrients and micronutrients, not all nutrients were reported equally in Canada’s overall food supply for infants and toddlers. Calcium, iron, potassium, and sodium were recorded by over 90% of the products. On the other hand, choline, copper, iodine, magnesium, manganese, phosphorus, and selenium were reported by fewer than 10%. This discrepancy might reflect differing priorities or perceptions regarding the importance of certain nutrients in the formulation of infant food products.

Of note, discrepancies in statistical significance between absolute nutrient values and their corresponding %DVs may arise from differences in reference standards used to calculate %DVs. These standards are based on age-specific daily requirements established by regulatory bodies such as Health Canada. For example, a product may contain a relatively small absolute amount of a nutrient, yet still display a high %DV if the recommended intake for that nutrient is low—common for micronutrients such as vitamin B12 or vitamin A in early childhood. Such differences highlight the importance of interpreting %DVs in the context of the reference population and not solely in comparison to absolute nutrient amounts.

The most frequently mentioned claim, ‘No added sugar’, underscores the industry’s focus on reducing sugar in infant foods. Yet, previous studies have shown that up to 60% of infants are introduced to solid foods and beverages containing added sugars^(34)^. In our sample, such claims were predominantly found on products from the United States, suggesting that labelling practices may be influenced by U.S. regulatory requirements, where the declaration of added sugars is mandatory. This may create a perception of greater transparency or healthfulness, although the presence of such claims does not always align with actual product composition. Despite being approximately four times higher in the older age group, sodium levels in these products remained well below the daily recommended upper limit of 1,200 mg for children aged one to four years^(12)^. Alignment with recommended thresholds may help explain the frequent appearance of the claim ‘No added sodium/salt’—the second most cited in our sample—indicating a strategic emphasis by manufacturers on highlighting sodium reduction in infant food marketing. These claims and actual reductions are important since exposure to tastes such as sweetness, saltiness, or fattiness during infancy can induce unhealthy food preferences that persist into adulthood^(35)^.

In our study, disease reduction claims were exceedingly rare, with only one product that included the following statement: ‘Various studies suggest that blueberries have the potential to stave off cognitive decline and support memory function’. While this product was targeted to infants six months of age and older, the Canadian Food Inspection Agency prohibits disease risk reduction claims on foods intended solely for children under four years old^(15)^, ensuring that parents are not misled by unsubstantiated health claims. The rarity of such claims highlights the industry’s adherence to regulatory standards and the emphasis on credible nutritional information.

Although several comparisons yielded statistically significant differences, it is important to recognise that not all of them may be of clinical or public health relevance. For instance, the difference in trans fat content between plant-based and non-plant-based products was statistically significant (*p* < 0.001); however, the mean values were 0.00 g and 0.004 g per 100 g, respectively—amounts that are likely of minimal nutritional concern and pose limited potential for harm in the context of typical infant consumption patterns. Nonetheless, it is worth highlighting that the World Health Organization recommends replacing industrially produced trans-fatty acids with unsaturated fats from plant sources and emphasises that such products should largely be avoided, underscoring the importance of continued surveillance even when quantities are minimal^(15)^.

This study offers a comprehensive assessment of the nutritional composition of commercially available infant foods in Canada, encompassing a broad spectrum of product categories and labelling claims. A key strength lies in the extensive dataset and systematic classification of products based on health-related claims, enabling nuanced analyses of nutritional composition, labelling, and market trends. The systematic and standardised approach to data extraction and categorisation further strengthens the reliability of comparisons across subgroups. In addition, nutrient values were standardised per 100 g or 100 mL, allowing for meaningful comparison across diverse product types and formats. However, several limitations warrant consideration. Nutrient data were derived from product labels rather than laboratory-based biochemical analyses, and labelling inconsistencies—such as missing values or non-standardised percent DVs—may have influenced the precision of nutrient estimates. The inclusion of added sugars data, for example, depended on manufacturer disclosure, which is not mandated in Canada and may not reflect uniform practices across all products. Another limitation is the potential misalignment between product marketing (e.g. age targets or claims like ‘for all ages’) and actual nutritional suitability, which may have affected how products were categorised for analysis. While we applied consistent criteria, some consumer-facing labels may introduce ambiguity. Finally, as a cross-sectional analysis, this study does not capture actual consumption patterns or dietary intake. Future research would benefit from linking product composition to feeding behaviours and nutritional outcomes to better understand the implications for infant and toddler health.

The significant differences in the nutritional composition of commercial infant foods across product categories underscore the importance of selecting foods and beverages based on both dietary needs and overall nutritional content. Of note, while the quantity of nutrients—such as calories, protein, fat, vitamins, and minerals—is important, the quality, balance, and bioavailability of these nutrients are equally critical to support healthy growth and development. This study contributes to the growing body of evidence on infant nutrition, offering relevant insights for parents, caregivers, and healthcare professionals.

Importantly, our findings have policy and industry implications. A considerable proportion of products contained high levels of total sugars, supporting the need for stronger FOP labelling regulations, such as ‘high in sugar’ warnings, to guide healthier choices. Additionally, observed deficiencies in certain micronutrients within specific product categories point to opportunities for the food industry to optimise formulations—particularly through targeted fortification—to better align with infants’ developmental needs. Future research should (1) examine how differences in nutrient content across products affect long-term health, (2) evaluate whether policy changes and product reformulations promote healthier feeding practices in early childhood, and (3) characterise actual dietary intake patterns. To help contextualise such findings, our laboratory is currently characterising dietary intake in infants and toddlers in Canada and assessing its alignment with nutritional guidelines.

## Supporting information

Fernando Ceccon and Kebbe supplementary material Fernando Ceccon and Kebbe supplementary material

## Supplementary material

To view supplementary material for this article, please visit https://doi.org/10.1017/jns.2025.10037.
