# Supplementary material for: Characterising nutritional composition and labelling of packaged infant foods in Canada
Source: J Nutr Sci. 2025 Sep 11;14:e64. doi: 10.1017/jns.2025.10037 (PMC12451243; doi:10.1017/jns.2025.10037)
Supplement: Fernando Ceccon and Kebbe supplementary material [file S2048679025100372sup001.docx]

**SUPPLEMENTARY MATERIAL**


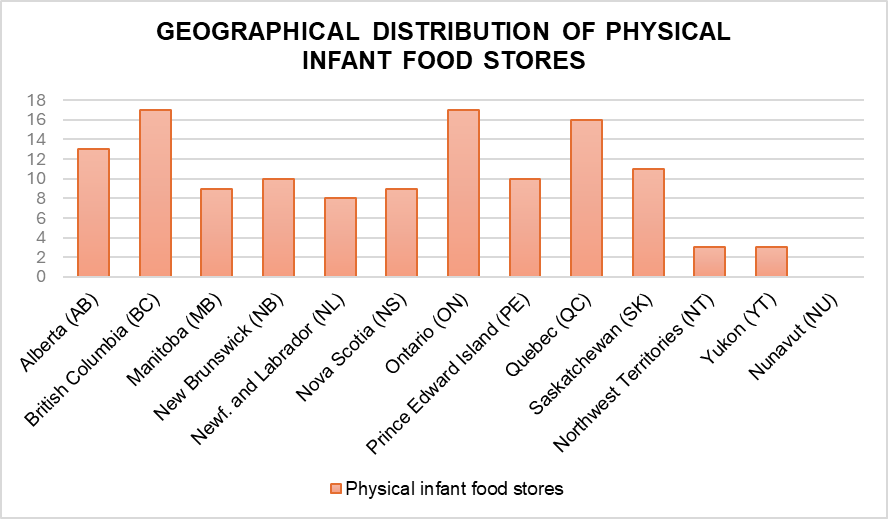
**Supplementary Figure 1**: Geographical distribution of physical infant food stores. Out of the 50 included stores, 38 operated physical locations, while 12 offered products exclusively through online sales.

**Supplementary Table 1. Extended Comparative Analysis of Products for Infants Under 12 Months and 12 Months or Older**

|  | 4-11 months¹ | n | 12-18 months | n | T-test |
| --- | --- | --- | --- | --- | --- |
| Serving size (g or mL) | 100.00 | 554 | 100.00 | 135 | - |
| Calories (kcal) | 139.92 ± 142.52 | 554 | 280.91 ± 161.99 | 135 | t(188.6) = 9.23, p<0.001 |
| Fat (g) | 2.26 ± 4.19 | 554 | 6.78 ± 7.23 | 135 | t(156.58) = 6.99, p<0.001 |
| *Saturated fat (g)* | 0.33 ± 0.92 | 355 | 1.12 ± 1.98 | 116 | t(131.57) = 4.11, p<0.001 |
| *Monounsaturated fat (g)* | 3.29 ± 0.36 | 2 | 0.09 | 1 | - |
| *Polyunsaturated fat (g)* | 0.83 ± 0.31 | 3 | 0.09 | 1 | - |
| *Omega-3 (g)* | - | 0 | 0.19 ± 0.08 | 3 | - |
| *Omega-6 (g)* | - | 0 | 0.35 | 1 | - |
| *Trans fat (g)* | 0.00 | 319 | 0.01 ± 0.02 | 114 | t(113.53) = 2.43, p = 0.017 |
| *Cholesterol (mg)* | 13.32 ± 13.55 | 26 | 9.10 ± 5.47 | 19 | t(34.98) = -1.44, p = 0.159 |
| Carbohydrates (g) | 26.69 ± 25.65 | 554 | 50.60 ± 29.67 | 135 | t(185.73) = 8.60, p<0.001 |
| *Sugar (g)* | 10.22 ± 9.97 | 552 | 17.52 ± 18.62 | 135 | t(153.25) = 4.41, p<0.001 |
| *Added sugar (g)* | 1.32 ± 2.26 | 17 | 16.94 ± 14.60 | 15 | t(14.59) = 4.1, p<0.001 |
| Fiber (g) | 2.51 ± 2.86 | 516 | 3.37 ± 4.89 | 131 | t(153.17) = 1.93, p = 0.056 |
| Protein (g) | 3.39 ± 4.43 | 554 | 6.03 ± 4.87 | 135 | t(195.65) = 5.75, p<0.001 |
| Calcium (mg) | 50.60 ± 122.92 | 496 | 87.31 ± 142.14 | 121 | t(166.43) = 2.61, p = 0.010 |
| Calcium (%) | 18.67 ± 45.89 | 496 | 12.48 ± 20.93 | 121 | t(424.95) = -2.21, p = 0.028 |
| Magnesium (mg) | 100.66 ± 135.32 | 37 | 43.04 ± 23.28 | 9 | t(42.46) = -2.45, p = 0.019 |
| Magnesium (%) | 134.06 ± 180.64 | 37 | 55.41 ± 31.81 | 9 | t(42.65) = -2.49, p = 0.017 |
| Phosphorus (mg) | 349.43 ± 173.47 | 13 | 184.10 ± 55.16 | 3 | t(11.54) = -2.87, p = 0.015 |
| Phosphorus (%) | 126.67 ± 63.44 | 13 | 31.53 ± 24.40 | 3 | t(9.32) = -4.22, p = 0.002 |
| Potassium (mg) | 223.73 ± 176.04 | 491 | 308.55 ± 253.32 | 117 | t(143.78) = 3.43, p<0.001 |
| Potassium (%) | 29.99 ± 23.88 | 491 | 12.02 ± 9.40 | 117 | t(479.05) = -12.99, p<0.001 |
| Sodium (mg) | 27.41 ± 54.30 | 554 | 108.94 ± 131.46 | 135 | t(145.31) = 7.06, p<0.001 |
| Choline (mg) | 289.98 ± 208.29 | 10 | - | 0 | - |
| Choline (%) | 184.75 ± 131.03 | 10 | - | 0 | - |
| Copper (mg) | 0.24 ± 0.20 | 4 | - | 0 | - |
| Copper (%) | 120.00 ± 103.32 | 4 | - | 0 | - |
| Iron (mg) | 3.54 ± 8.96 | 497 | 4.97 ± 9.91 | 127 | t(182.11) = 1.47, p = 0.142 |
| Iron (%) | 33.20 ± 82.58 | 497 | 70.70 ± 142.07 | 127 | t(148.41) = 2.85, p = 0.005 |
| Iodide (ug) | 28.39 ± 53.81 | 24 | 16.29 ± 2.74 | 5 | t(23.55) = -1.10, p = 0.285 |
| Iodide (%) | 22.01 ± 41.20 | 24 | 17.15 ± 3.91 | 5 | t(24.77) = -0.57, p = 0.576 |
| Manganese (mg) | 1.56 ± 1.91 | 6 | 1.01 ± 0.77 | 4 | t(7.05) = -0.63, p = 0.546 |
| Manganese (%) | 79.87 ± 79.30 | 6 | 76.38 ± 56.57 | 4 | t(7.88) = -0.08, p = 0.937 |
| Selenium (mcg) | 2.02 | 2 | 14.29 | 3 | - |
| Selenium (%) | 10.10 | 2 | 71.43 | 3 | - |
| Zinc (mg) | 3.32 ± 3.69 | 44 | 1.91 ± 1.73 | 16 | t(53.97) = -1.99, p = 0.051 |
| Zinc (%) | 110.37 ± 122.06 | 44 | 65.35 ± 60.39 | 16 | t(552.36) = -1.89, p = 0.064 |
| Vitamin A (ug) | 218.66 ± 381.12 | 107 | 79.91 ± 89.42 | 18 | t(111.96) = -3.27, p = 0.001 |
| Vitamin A (%) | 45.91 ± 74.64 | 107 | 26.60 ± 30.47 | 18 | t(59) = -1.90, p = 0.063 |
| Vitamin B1 (mg) | 1.13 ± 0.61 | 56 | 1.60 ± 1.49 | 20 | t(21.29) = 1.37, p = 0.184 |
| Vitamin B1 (%) | 374.73 ± 202.68 | 56 | 316.01 ± 300.14 | 20 | t(25.46) = -0.81, p = 0.425 |
| Vitamin B2 (mg) | 1.56 ± 0.66 | 36 | 1.18 ± 0.93 | 13 | t(16.60) = -1.35, p = 0.195 |
| Vitamin B2 (%) | 392.04 ± 165.13 | 36 | 236.69 ± 185.24 | 13 | t(19.33) = -2.67, p = 0.015 |
| Vitamin B3 (mg) | 12.97 ± 7.66 | 43 | 15.73 ± 15.57 | 21 | t(24.84) = 0.77, p = 0.451 |
| Vitamin B3 (%) | 325.86 ± 191.25 | 43 | 257.88 ± 263.04 | 21 | t(30.69) = -1.06, p = 0.299 |
| Vitamin B5 (mg) | 2.11 ± 1.38 | 11 | 0.43 | 2 | t(10) = -4.05, p = 0.002 |
| Vitamin B5 (%) | 116.02 ± 77.03 | 11 | 21.43 | 2 | t(10) = -4.07, p = 0.002 |
| Vitamin B6 (mg) | 0.55 ± 1.62 | 27 | 0.34 ± 0.20 | 9 | t(28.20) = -0.65, p = 0.522 |
| Vitamin B6 (%) | 82.96 ± 66.62 | 27 | 68.04 ± 39.15 | 9 | t(24.01) = -0.82, p = 0.423 |
| Vitamin B7 (ug) | 6.93 ± 6.99 | 28 | 6.32 ± 3.28 | 10 | t(32.98) = -0.37, p = 0.716 |
| Vitamin B7 (%) | 116.44 ± 117.16 | 28 | 79.44 ± 40.17 | 10 | t(36) = -1.45, p = 0.156 |
| Vitamin B9 (ug) | 31.33 ± 33.68 | 22 | 42.32 ± 60.74 | 4 | t(3.34) = 0.35, p = 0.746 |
| Vitamin B9 (%) | 67.27 ± 59.61 | 22 | 40.55 ± 61.86 | 4 | t(4.08) = 0.80, p = 0.468 |
| Vitamin B12 (ug) | 0.33 ± 0.42 | 22 | 13.69 ± 42.04 | 10 | t(9) = 1.00, p = 0.341 |
| Vitamin B12 (%) | 64.50 ± 83.11 | 22 | 51.62 ± 31.46 | 10 | t(29.49) = -0.63, p = 0.531 |
| Vitamin C (mg) | 24.44 ± 23.71 | 108 | 11.68 ± 7.75 | 28 | t(127.53) = -4.71, p<0.001 |
| Vitamin C (%) | 48.85 ± 47.21 | 108 | 78.26 ± 52.69 | 28 | t(38.98) = 2.69, p = 0.011 |
| Vitamin D (ug) | 5.84 ± 5.22 | 24 | 2.38 ± 2.61 | 6 | t(16.44) = -2.30, p = 0.035 |
| Vitamin D (%) | 58.41 ± 52.12 | 24 | 14.29 ± 15.65 | 6 | t(26.64) = -3.56, p = 0.001 |
| Vitamin E (mg) | 4.34 ± 4.25 | 44 | 4.23 ± 2.70 | 15 | t(38.68) = -0.11, p = 0.913 |
| Vitamin E (%) | 84.51 ± 82.54 | 44 | 63.96 ± 44.13 | 15 | t(46) = -1.22, p = 0.229 |
| Vitamin K (ug) | 6.01 ± 10.62 | 19 | 23.50 ± 13.50 | 4 | t(3.82) = 2.44, p = 0.074 |
| Vitamin K (%) | 239.94 ± 424.94 | 19 | 80.70 ± 46.33 | 4 | t(19.71) = -1.59, p = 0.128 |

¹Values are presented as mean ± standard deviation.

**Supplementary Table 2. Comparative Analysis of Organic and Non-Organic Products**

|  | Organic¹ | n | Non-organic | n | T-test |
| --- | --- | --- | --- | --- | --- |
| Serving size (g or mL) | 100.00 | 728 | 100.00 | 282 | - |
| Calories (kcal) | 155.98 ± 152.06 | 728 | 193.26 ± 168.73 | 282 | t(467.71) = 3.24, p = 0.001 |
| Fat (g) | 2.58 ± 5.05 | 728 | 3.87 ± 5.80 | 282 | t(454.79) = 3.28, p = 0.001 |
| *Saturated fat (g)* | 0.48 ± 1.87 | 544 | 0.74 ± 1.56 | 226 | t(499.26) = 2.02, p = 0.043 |
| *Monounsaturated fat (g)* | 0.09 | 1 | 3.29 ± 0.36 | 2 | - |
| *Polyunsaturated fat (g)* | 0.58 ± 0.38 | 4 | 1.01 ± 0 | 2 | t(3) = 2.24, p = 0.111 |
| *Omega-3 (g)* | 0.19 ± 0.08 | 3 | - | 0 | - |
| *Omega-6 (g)* | 0.35 | 1 | - | 0 | - |
| *Trans fat (g)* | 0.00 ± 0.01 | 508 | 0.00 ± 0.02 | 222 | t(240.25) = 1.86, p = 0.065 |
| *Cholesterol (mg)* | 16.34 ± 29.65 | 21 | 11.20 ± 13.03 | 63 | t(22.63) = -0.77, p = 0.450 |
| Carbohydrates (g) | 30.41 ± 28.15 | 728 | 34.02 ± 31.38 | 282 | t(466) = 1.69, p = 0.092 |
| *Sugar (g)* | 11.23 ± 11.38 | 728 | 12.93 ± 14.72 | 282 | t(413.72) = 1.75, p = 0.081 |
| *Added sugar (g)* | 12.97 ± 14.31 | 39 | 4.87 ± 5.77 | 13 | t(47.98) = -2.90, p = 0.006 |
| Fiber (g) | 3.01 ± 3.59 | 699 | 1.99 ± 3.52 | 269 | t(495.58) = -4.04, p<0.001 |
| Protein (g) | 3.49 ± 4.71 | 728 | 4.86 ± 4.89 | 282 | t(494.47) = 4.03, p<0.001 |
| Calcium (mg) | 51.05 ± 124.00 | 664 | 92.38 ± 137.17 | 266 | t(447.73) = 4.27, p<0.001 |
| Calcium (%) | 16.18 ± 43.97 | 664 | 24.60 ± 46.67 | 266 | t(463.54) = 2.43, p = 0.012 |
| Magnesium (mg) | 72.92 ± 123.80 | 47 | 68.62 ± 53.60 | 28 | t(68.03) = -0.21, p = 0.836 |
| Magnesium (%) | 96.56 ± 164.91 | 47 | 90.56 ± 71.64 | 28 | t(68.12) = -0.22, p = 0.829 |
| Phosphorus (mg) | 261.14 ± 174.98 | 14 | 365.65 ± 136.75 | 11 | t(23) = 1.68, p = 0.107 |
| Phosphorus (%) | 93.53 ± 67.47 | 14 | 127.50 ± 57.38 | 11 | t(22.82) = 1.36, p = 0.187 |
| Potassium (mg) | 241.03 ± 206.58 | 661 | 240.19 ± 230.62 | 254 | t(419.01) = -0.05, p = 0.959 |
| Potassium (%) | 25.84 ± 24.35 | 661 | 22.40 ± 27.23 | 254 | t(417.32) = -1.76, p = 0.080 |
| Sodium (mg) | 31.34 ± 65.94 | 728 | 73.65 ± 97.55 | 282 | t(384.6) = 6.71, p<0.001 |
| Choline (mg) | 145.97 ± 165.18 | 38 | 428.57 | 3 | t(37) = 10.55, p<0.001 |
| Choline (%) | 88.10 ± 99.59 | 38 | 285.71 | 3 | t(37) = 12.23, p<0.001 |
| Copper (mg) | 0.23 ± 0.25 | 23 | 0.07 ± 0.04 | 4 | t(25) = -2.90, p = 0.008 |
| Copper (%) | 107.43 ± 117.06 | 23 | 33.33 ± 19.44 | 4 | t(24.93) = -2.82, p = 0.009 |
| Iron (mg) | 2.97 ± 8.26 | 671 | 5.98 ± 11.64 | 263 | t(369.9) = 3.83, p<0.001 |
| Iron (%) | 30.75 ± 84.20 | 671 | 59.37 ± 116.35 | 263 | t(374.42) = 3.63, p<0.001 |
| Iodide (ug) | 33.66 ± 49.78 | 29 | 17.19 ± 20.49 | 27 | t(37.77) = -1.64, p = 0.110 |
| Iodide (%) | 29.08 ± 40.07 | 29 | 14.21 ± 15.85 | 27 | t(37.08) = -1.85, p = 0.072 |
| Manganese (mg) | 0.25 ± 0.49 | 29 | 8.33 ± 6.00 | 4 | t(3.01) = 2.69, p = 0.074 |
| Manganese (%) | 29.42 ± 53.84 | 29 | 41.67 ± 30.00 | 4 | t(6.13) = 0.68, p = 0.522 |
| Selenium (mcg) | 10.74 ± 12.64 | 26 | - | 0 | - |
| Selenium (%) | 49.47 ± 60.41 | 26 | - | 0 | - |
| Zinc (mg) | 3.32 ± 3.11 | 55 | 2.35 ± 3.15 | 58 | t(110.83) = -1.65, p = 0.102 |
| Zinc (%) | 111.50 ± 99.06 | 55 | 76.54 ± 102.36 | 58 | t(110.95) = -1.85, p = 0.068 |
| Vitamin A (ug) | 220.01 ± 339.87 | 122 | 151.00 ± 244.41 | 59 | t(153.04) = -1.56, p = 0.121 |
| Vitamin A (%) | 50.61 ± 70.32 | 122 | 27.14 ± 39.94 | 59 | t(174.39) = -2.86, p = 0.005 |
| Vitamin B1 (mg) | 1.08 ± 1.07 | 57 | 1.05 ± 0.59 | 48 | t(89.75) = -0.18, p = 0.857 |
| Vitamin B1 (%) | 306.82 ± 254.07 | 57 | 317.25 ± 188.83 | 48 | t(101.52) = 0.24, p = 0.810 |
| Vitamin B2 (mg) | 1.10 ± 0.76 | 37 | 1.43 ± 0.75 | 40 | t(74.47) = 1.92, p = 0.059 |
| Vitamin B2 (%) | 269.11 ± 189.90 | 37 | 342.30 ± 175.11 | 40 | t(73.50) = 1.74, p = 0.086 |
| Vitamin B3 (mg) | 8.66 ± 10.52 | 65 | 12.98 ± 7.90 | 47 | t(109.82) = 2.48, p = 0.015 |
| Vitamin B3 (%) | 185.28 ± 196.14 | 65 | 303.69 ± 190.34 | 47 | t(100.97) = 3.21, p = 0.002 |
| Vitamin B5 (mg) | 4.80 ± 1.10 | 9 | 0.99 ± 0.66 | 12 | t(12.21) = -9.21, p<0.001 |
| Vitamin B5 (%) | 247.62 ± 50.95 | 9 | 54.37 ± 37.02 | 12 | t(13.99) = -9.63, p<0.001 |
| Vitamin B6 (mg) | 0.28 ± 0.20 | 58 | 0.67 ± 1.78 | 22 | t(21.20) = 1.03, p = 0.314 |
| Vitamin B6 (%) | 80.85 ± 54.77 | 58 | 97.48 ± 76.50 | 22 | t(29.55) = 0.93, p = 0.358 |
| Vitamin B7 (ug) | 5.14 ± 4.59 | 16 | 6.38 ± 6.76 | 29 | t(40.96) = 0.73, p = 0.469 |
| Vitamin B7 (%) | 78.12 ± 72.72 | 16 | 102.59 ± 112.94 | 29 | t(41.82) = 0.88, p = 0.383 |
| Vitamin B9 (ug) | 57.25 ± 75.42 | 46 | 23.56 ± 31.72 | 21 | t(64.75) = -2.57, p = 0.012 |
| Vitamin B9 (%) | 78.81 ± 95.39 | 46 | 51.52 ± 41.69 | 21 | t(64.95) = -1.63, p = 0.108 |
| Vitamin B12 (ug) | 0.55 ± 0.52 | 47 | 5.20 ± 25.61 | 27 | t(26.01) = 0.94, p = 0.354 |
| Vitamin B12 (%) | 92.71 ± 93.74 | 47 | 55.32 ± 59.99 | 27 | t(71.07) = -2.09, p = 0.040 |
| Vitamin C (mg) | 22.95 ± 20.08 | 128 | 22.41 ± 24.45 | 70 | t(120.39) = -0.16, p = 0.875 |
| Vitamin C (%) | 55.78 ± 45.08 | 128 | 53.08 ± 50.27 | 70 | t(129.44) = -0.37, p = 0.709 |
| Vitamin D (ug) | 4.72 ± 4.77 | 53 | 1.94 ± 1.70 | 48 | t(66.08) = -3.97, p<0.001 |
| Vitamin D (%) | 45.40 ± 45.54 | 53 | 13.43 ± 18.61 | 48 | t(70.31) = -4.70, p<0.001 |
| Vitamin E (mg) | 4.41 ± 3.80 | 59 | 4.18 ± 4.00 | 46 | t(94.39) = -0.29, p = 0.773 |
| Vitamin E (%) | 81.19 ± 69.54 | 59 | 76.69 ± 76.55 | 46 | t(91.99) = -0.31, p = 0.757 |
| Vitamin K (ug) | 6.49 ± 9.99 | 21 | 36.00 ± 1.41 | 2 | t(15.54) = 12.31, p<0.001 |
| Vitamin K (%) | 95.31 ± 42.09 | 21 | 1,440.00 ± 56.57 | 2 | t(1.11) = 32.76, p = 0.014 |

¹Values are presented as mean ± standard deviation.

**Supplementary Table 3. Comparative Analysis of Plant-Based and Non-Plant-Based Products**

|  | Plant-based¹ | n | Non-plant-based | n | T-test |
| --- | --- | --- | --- | --- | --- |
| Serving size (g or mL) | 100.00 | 750 | 100.00 | 260 | - |
| Calories (kcal) | 155.72 ± 151.31 | 750 | 197.17 ± 171.44 | 260 | t(407.48) = 3.46, p<0.001 |
| Fat (g) | 2.11 ± 4.55 | 750 | 5.32 ± 6.45 | 260 | t(352.40) = 7.40, p<0.001 |
| *Saturated fat (g)* | 0.36 ± 1.76 | 581 | 1.16 ± 1.73 | 189 | t(325.08) = 5.49, p<0.001 |
| *Monounsaturated fat (g)* | 0.09 | 1 | 3.29 ± 0.36 | 2 | - |
| *Polyunsaturated fat (g)* | 0.58 ± 0.38 | 4 | 1.01 | 2 | t(3.00) = 2.24, p = 0.111 |
| *Omega-3 (g)* | 0.19 ± 0.08 | 3 | - | 0 | - |
| *Omega-6 (g)* | 0.35 | 1 | - | 0 | - |
| *Trans fat (g)* | 0.00 | 50 | 0.004 ± 0.02 | 180 | t(179.00) = 2.96, p = 0.004 |
| *Cholesterol (mg)* | - | 0 | 12.48 ± 18.54 | 84 | - |
| Carbohydrates (g) | 31.74 ± 28.94 | 750 | 30.50 ± 29.67 | 260 | t(441.37) = -0.59, p = 0.559 |
| *Sugar (g)* | 11.64 ± 11.29 | 750 | 11.87 ± 15.25 | 260 | t(358.62) = 0.22, p = 0.823 |
| *Added sugar (g)* | 9.86 ± 13.16 | 33 | 12.84 ± 13.27 | 19 | t(37.42) = 0.78, p = 0.440 |
| Fiber (g) | 3.04 ± 3.85 | 718 | 1.82 ± 2.56 | 250 | t(654.69) = -5.65, p < 0.001 |
| Protein (g) | 2.96 ± 4.25 | 750 | 6.49 ± 5.30 | 260 | t(380.93) = 9.70, p < 0.001 |
| Calcium (mg) | 41.80 ± 108.59 | 681 | 120.49 ± 160.11 | 249 | (t(334.97) = 7.18, p < 0.001 |
| Calcium (%) | 13.54 ± 38.74 | 681 | 32.38 ± 56.34 | 249 | t(337.49) = 4.87, p < 0.001 |
| Magnesium (mg) | 77.76 ± 128.36 | 43 | 62.65 ± 52.86 | 32 | t(59.16) = -0.70, p = 0.489 |
| Magnesium (%) | 103.07 ± 170.99 | 43 | 82.56 ± 70.61 | 32 | t(59.24) = -0.71, p = 0.481 |
| Phosphorus (mg) | 290.65 ± 170.53 | 14 | 328.09 ± 162.98 | 11 | t(22.05) = 0.56, p = 0.582 |
| Phosphorus (%) | 104.81 ± 65.39 | 14 | 113.16 ± 65.73 | 11 | t(21.57) = 0.32, p = 0.755 |
| Potassium (mg) | 237.34 ± 212.57 | 678 | 250.61 ± 216.00 | 237 | t(407.77) = 0.82, p = 0.415 |
| Potassium (%) | 25.11 ± 23.53 | 678 | 24.25 ± 29.56 | 237 | t(346.26) = -0.40, p = 0.686 |
| Sodium (mg) | 22.06 ± 50.01 | 750 | 104.00 ± 108.15 | 260 | t(298.26) = 11.79, p < 0.001 |
| Choline (mg) | 178.67 ± 176.80 | 38 | 14.45 ± 3.68 | 3 | t(37.39) = -5.71, p < 0.001 |
| Choline (%) | 109.88 ± 109.99 | 38 | 9.73 ± 3.19 | 3 | t(37.71) = -5.58, p < 0.001 |
| Copper (mg) | 0.24 ± 0.27 | 20 | 0.10 ± 0.08 | 7 | t(24.81) = -2.07, p = 0.049 |
| Copper (%) | 112.48 ± 124.04 | 20 | 50.65 ± 39.06 | 7 | t(24.95) = -1.97, p = 0.060 |
| Iron (mg) | 3.63 ± 9.48 | 690 | 4.34 ± 9.26 | 244 | t(435.48) = 1.03, p = 0.305 |
| Iron (%) | 36.03 ± 93.34 | 690 | 46.67 ± 99.97 | 244 | t(402.44) = 1.45, p = 0.147 |
| Iodide (ug) | 33.42 ± 52.84 | 26 | 19.04 ± 19.86 | 30 | t(31.10) = -1.31, p = 0.200 |
| Iodide (%) | 28.99 ± 42.58 | 26 | 15.77 ± 15.34 | 30 | t(30.60) = -1.50, p = 0.144 |
| Manganese (mg) | 0.28 ± 0.51 | 26 | 4.76 ± 6.15 | 7 | t(6.02) = 1.93, p = 0.102 |
| Manganese (%) | 32.81 ± 55.95 | 26 | 23.81 ± 30.76 | 7 | t(18.02) = -0.56, p = 0.580 |
| Selenium (mcg) | 10.45 ± 12.76 | 23 | 12.98 ± 14.11 | 3 | t(2.45) = 0.29, p = 0.792 |
| Selenium (%) | 52.26 ± 63.79 | 23 | 28.02 ± 9.21 | 3 | t(23.10) = -1.69, p = 0.104 |
| Zinc (mg) | 3.94 ± 3.64 | 66 | 1.24 ± 1.04 | 47 | t(79.39) = -5.71, p < 0.001 |
| Zinc (%) | 130.39 ± 117.09 | 66 | 41.83 ± 34.63 | 47 | t(80.24) = -5.80, p < 0.001 |
| Vitamin A (ug) | 211.24 ± 334.79 | 123 | 168.41 ± 261.02 | 58 | t(140.29) = -0.94, p = 0.350 |
| Vitamin A (%) | 48.29 ± 69.50 | 123 | 31.66 ± 44.43 | 58 | t(163.04) = -1.94, p = 0.054 |
| Vitamin B1 (mg) | 0.98 ± 1.05 | 63 | 1.19 ± 0.52 | 42 | t(96.05) = 1.38, p = 0.172 |
| Vitamin B1 (%) | 284.94 ± 250.78 | 63 | 351.55 ± 176.86 | 42 | t(102.63) = 1.60, p = 0.114 |
| Vitamin B2 (mg) | 0.97 ± 0.72 | 46 | 1.71 ± 0.61 | 31 | t(70.82) = 4.83, p < 0.001 |
| Vitamin B2 (%) | 240.53 ± 181.96 | 46 | 405.97 ± 146.24 | 31 | t(72.58) = 4.41, p < 0.001 |
| Vitamin B3 (mg) | 8.45 ± 9.96 | 72 | 14.12 ± 8.16 | 40 | t(94.62) = 3.25, p = 0.0016 |
| Vitamin B3 (%) | 186.79 ± 186.49 | 72 | 321.70 ± 200.89 | 40 | t(75.75) = 3.49, p < 0.001 |
| Vitamin B5 (mg) | 4.38 ± 1.69 | 10 | 1.02 ± 0.68 | 11 | t(11.62) = -5.88, p < 0.001 |
| Vitamin B5 (%) | 226.19 ± 83.06 | 10 | 56.28 ± 38.20 | 11 | t(12.38) = -5.92, p < 0.001 |
| Vitamin B6 (mg) | 0.42 ± 1.03 | 67 | 0.20 ± 0.10 | 13 | t(71.62) = -1.71, p = 0.092 |
| Vitamin B6 (%) | 90.40 ± 64.67 | 67 | 59.77 ± 31.72 | 13 | t(35.02) = -2.59, p = 0.014 |
| Vitamin B7 (ug) | 4.70 ± 4.73 | 16 | 6.63 ± 6.65 | 29 | t(40.05) = 1.13, p = 0.264 |
| Vitamin B7 (%) | 72.54 ± 75.19 | 16 | 105.67 ± 111.25 | 29 | t(41.04) = 1.19, p = 0.242 |
| Vitamin B9 (ug) | 50.37 ± 70.85 | 53 | 32.75 ± 46.39 | 14 | t(31.02) = -1.12, p = 0.272 |
| Vitamin B9 (%) | 77.46 ± 90.72 | 53 | 43.00 ± 32.69 | 14 | t(58.82) = -2.26, p = 0.027 |
| Vitamin B12 (ug) | 3.18 ± 18.60 | 51 | 0.18 ± 0.14 | 23 | t(50.01) = -1.15, p = 0.255 |
| Vitamin B12 (%) | 102.47 ± 92.40 | 51 | 27.16 ± 15.01 | 23 | t(55.59) = -5.66, p < 0.001 |
| Vitamin C (mg) | 23.42 ± 19.52 | 161 | 19.86 ± 29.40 | 37 | t(43.56) = -0.70, p = 0.486 |
| Vitamin C (%) | 55.45 ± 43.19 | 161 | 52.11 ± 61.00 | 37 | t(44.64) = -0.31, p = 0.755 |
| Vitamin D (ug) | 4.92 ± 4.95 | 47 | 2.08 ± 1.87 | 54 | t(57.41) = -3.71, p < 0.001 |
| Vitamin D (%) | 46.98 ± 47.27 | 47 | 15.60 ± 20.52 | 54 | t(60.91) = -4.22, p < 0.001 |
| Vitamin E (mg) | 4.78 ± 4.05 | 67 | 3.48 ± 3.43 | 38 | t(87.71) = 1.75, p = 0.084 |
| Vitamin E (%) | 87.64 ± 74.08 | 67 | 64.38 ± 67.67 | 38 | t(82.92) = -1.63, p = 0.106 |
| Vitamin K (ug) | 6.69 ± 10.21 | 20 | 24.84 ± 19.35 | 3 | t(2.17) = 1.59, p = 0.243 |
| Vitamin K (%) | 95.03 ± 43.16 | 20 | 993.67 ± 774.10 | 3 | t(2.00) = 2.01, p = 0.182 |

¹Values are presented as mean ± standard deviation.

**Supplementary Table 4. Comparative Analysis of Vegetarian/Vegan and Non-Vegetarian/Vegan Products**

|  | Vegetarian/vegan¹ | n | Non-veg | n | T-test |
| --- | --- | --- | --- | --- | --- |
| Serving size (g or mL) | 100.00 | 747 | 100.00 | 263 | - |
| Calories (kcal) | 155.70 ± 151.27 | 747 | 196.76 ± 171.36 | 263 | t(414.51) = 3.44, p < 0.001 |
| Fat (g) | 2.12 ± 4.56 | 747 | 5.27 ± 6.44 | 263 | t(358.57) = 7.30, p < 0.001 |
| *Saturated fat (g)* | 0.36 ± 1.76 | 581 | 1.15 ± 1.73 | 189 | t(324.87) = 5.41, p < 0.001 |
| *Monounsaturated fat (g)* | 0.09 | 1 | 3.29 ± 0.36 | 2 | - |
| *Polyunsaturated fat (g)* | 0.58 ± 0.38 | 4 | 1.01 | 2 | t(3.00) = 2.24, p = 0.111 |
| *Omega-3 (g)* | 0.19 ± 0.08 | 3 | - | 0 | - |
| *Omega-6 (g)* | 0.35 | 1 | - | 0 | - |
| *Trans fat (g)* | 0.00 | 550 | 0.004 ± 0.02 | 180 | t(179.00) = 2.96, p = 0.003 |
| *Cholesterol (mg)* | 126.26 | 1 | 11.11 ± 13.71 | 83 | - |
| Carbohydrates (g) | 31.72 ± 28.96 | 747 | 30.57 ± 29.59 | 263 | t(450.48) = -0.54, p = 0.587 |
| *Sugar (g)* | 11.63 ± 11.31 | 747 | 11.90 ± 15.16 | 263 | t(366.24) = 0.27, p = 0.789 |
| *Added sugar (g)* | 9.86 ± 13.16 | 33 | 12.84 ± 13.27 | 19 | t(37.42) = 0.78, p = 0.440 |
| Fiber (g) | 3.04 ± 3.85 | 715 | 1.85 ± 2.57 | 253 | t(664.81) = -5.51, p < 0.001 |
| Protein (g) | 2.96 ± 4.25 | 747 | 6.46 ± 5.32 | 263 | t(385.05) = 9.62, p < 0.001 |
| Calcium (mg) | 41.13 ± 107.00 | 678 | 121.36 ± 161.81 | 252 | t(335.93) = 7.30, p < 0.001 |
| Calcium (%) | 13.27 ± 38.05 | 678 | 32.38 ± 57.22 | 252 | t(336.93) = 5.04, p < 0.001 |
| Magnesium (mg) | 77.76 ± 128.36 | 43 | 62.65 ± 52.86 | 32 | t(59.16) = -0.70, p = 0.489 |
| Magnesium (%) | 103.07 ± 170.99 | 43 | 82.56 ± 70.61 | 32 | t(59.24) = -0.71, p = 0.481 |
| Phosphorus (mg) | 290.65 ± 170.53 | 14 | 328.09 ± 162.98 | 11 | t(22.05) = 0.56, p = 0.582 |
| Phosphorus (%) | 104.81 ± 65.39 | 14 | 113.16 ± 65.73 | 11 | t(21.57) = 0.32, p = 0.755 |
| Potassium (mg) | 237.50 ± 212.87 | 676 | 250.05 ± 215.17 | 239 | t(414.95) = 0.78, p = 0.438 |
| Potassium (%) | 25.10 ± 23.57 | 676 | 24.26 ± 29.43 | 239 | t(351.79) = -0.40, p = 0.691 |
| Sodium (mg) | 22.22 ± 50.19 | 747 | 102.62 ± 108.10 | 263 | t(302.67) = 11.63, p<0.001 |
| Choline (mg) | 178.67 ± 176.80 | 38 | 14.45 ± 3.68 | 3 | t(37.39) = -5.71, p < 0.001 |
| Choline (%) | 109.88 ± 109.99 | 38 | 9.73 ± 3.19 | 3 | t(37.71) = -5.58, p < 0.001 |
| Copper (mg) | 0.24 ± 0.27 | 20 | 0.10 ± 0.08 | 7 | t(24.81) = -2.07, p = 0.049 |
| Copper (%) | 112.48 ± 124.04 | 20 | 50.65 ± 39.06 | 7 | t(24.95) = -1.97, p = 0.060 |
| Iron (mg) | 3.59 ± 9.40 | 687 | 4.45 ± 9.48 | 247 | t(431.57) = 1.23, p = 0.220 |
| Iron (%) | 35.66 ± 92.72 | 687 | 47.56 ± 101.39 | 247 | t(403.11) = 1.62, p = 0.107 |
| Iodide (ug) | 33.42 ± 52.84 | 26 | 19.04 ± 19.86 | 30 | t(31.10) = -1.31, p = 0.200 |
| Iodide (%) | 28.99 ± 42.58 | 26 | 15.77 ± 15.34 | 30 | t(30.60) = -1.50, p = 0.144 |
| Manganese (mg) | 0.28 ± 0.51 | 26 | 4.76 ± 6.15 | 7 | t(6.02) = 1.93, p = 0.102 |
| Manganese (%) | 32.81 ± 55.95 | 26 | 23.81 ± 30.76 | 7 | t(18.02) = -0.56, p = 0.580 |
| Selenium (mcg) | 10.45 ± 12.76 | 23 | 12.98 ± 14.11 | 3 | t(2.45) = 0.30, p = 0.792 |
| Selenium (%) | 52.26 ± 63.79 | 23 | 28.02 ± 9.21 | 3 | t(23.10) = -1.69, p = 0.104 |
| Zinc (mg) | 3.94 ± 3.64 | 66 | 1.24 ± 1.04 | 47 | t(79.39) = -5.71, p < 0.001 |
| Zinc (%) | 130.39 ± 117.09 | 66 | 41.83 ± 34.63 | 47 | t(80.24) = -5.80, p < 0.001 |
| Vitamin A (ug) | 211.24 ± 334.79 | 123 | 168.41 ± 261.02 | 58 | t(140.29) = -0.94, p = 0.350 |
| Vitamin A (%) | 48.29 ± 69.50 | 123 | 31.66 ± 44.43 | 58 | t(163.04) = -1.94, p = 0.054 |
| Vitamin B1 (mg) | 0.98 ± 1.05 | 62 | 1.19 ± 0.51 | 43 | t(93.52) = 1.36, p = 0.178 |
| Vitamin B1 (%) | 283.78 ± 252.65 | 62 | 351.68 ± 174.74 | 43 | t(103) = 1.63, p = 0.107 |
| Vitamin B2 (mg) | 0.96 ± 0.73 | 45 | 1.70 ± 0.61 | 32 | t(72.96) = 4.87, p < 0.001 |
| Vitamin B2 (%) | 237.93 ± 183.16 | 45 | 404.44 ± 144.12 | 32 | t(74.17) = 4.46, p < 0.001 |
| Vitamin B3 (mg) | 8.37 ± 10.00 | 71 | 14.12 ± 8.16 | 41 | t(98.35) = 3.33, p = 0.001 |
| Vitamin B3 (%) | 184.39 ± 186.69 | 71 | 322.56 ± 198.44 | 41 | t(79.48) = 3.63, p < 0.001 |
| Vitamin B5 (mg) | 4.38 ± 1.69 | 10 | 1.02 ± 0.68 | 11 | t(11.62) = -5.89, p < 0.001 |
| Vitamin B5 (%) | 226.19 ± 83.06 | 10 | 56.28 ± 38.20 | 11 | t(12.38) = -5.92, p < 0.001 |
| Vitamin B6 (mg) | 0.42 ± 1.03 | 67 | 0.20 ± 0.10 | 13 | t(71.62) = -1.71, p = 0.092 |
| Vitamin B6 (%) | 90.40 ± 64.67 | 67 | 59.77 ± 31.72 | 13 | t(35.02) = -2.59, p = 0.014 |
| Vitamin B7 (ug) | 4.70 ± 4.73 | 16 | 6.63 ± 6.65 | 29 | t(40.05) = 1.13, p = 0.264 |
| Vitamin B7 (%) | 72.54 ± 75.19 | 16 | 105.67 ± 111.25 | 29 | t(41.04) = 1.19, p = 0.242 |
| Vitamin B9 (ug) | 50.37 ± 70.85 | 53 | 32.75 ± 46.39 | 14 | t(31.02) = -1.12, p = 0.272 |
| Vitamin B9 (%) | 77.46 ± 90.72 | 53 | 43.00 ± 32.69 | 14 | t(58.82) = -2.26, p = 0.027 |
| Vitamin B12 (ug) | 3.18 ± 18.60 | 51 | 0.18 ± 0.14 | 23 | t(50.01) = -1.15, p = 0.255 |
| Vitamin B12 (%) | 102.47 ± 92.40 | 51 | 27.16 ± 15.01 | 23 | t(55.59) = -5.66, p < 0.001 |
| Vitamin C (mg) | 23.42 ± 19.52 | 161 | 19.86 ± 29.40 | 37 | t(43.56) = -0.70, p = 0.486 |
| Vitamin C (%) | 55.45 ± 43.19 | 161 | 52.11 ± 61.00 | 37 | t(44.64) = -0.32, p = 0.755 |
| Vitamin D (ug) | 4.83 ± 4.93 | 48 | 2.10 ± 1.88 | 53 | t(59.27) = -3.60, p < 0.001 |
| Vitamin D (%) | 46.13 ± 47.14 | 48 | 15.78 ± 20.68 | 53 | t(63.08) = -4.12, p < 0.001 |
| Vitamin E (mg) | 4.78 ± 4.05 | 67 | 3.48 ± 3.43 | 38 | t(57.71) = -1.75, p = 0.084 |
| Vitamin E (%) | 87.64 ± 74.08 | 67 | 64.38 ± 67.67 | 38 | t(82.92) = -1.64, p = 0.106 |
| Vitamin K (ug) | 6.69 ± 10.21 | 20 | 24.84 ± 19.35 | 3 | t(2.17) = 1.59, p = 0.243 |
| Vitamin K (%) | 95.03 ± 43.16 | 20 | 993.67 ± 774.10 | 3 | t(2.00) = 2.01, p = 0.182 |

¹Values are presented as mean ± standard deviation.

**Supplementary Table 5. Comparative Analysis of Non-GMO Labeled and Unlabeled Products**

|  | Non-GMO Labeled¹ | n | Unlabeled | n | T-test |
| --- | --- | --- | --- | --- | --- |
| Serving size (g or mL) | 100.00 | 439 | 100.00 | 571 | - |
| Calories (kcal) | 184.19 ± 166.87 | 439 | 152.71 ± 148.97 | 571 | t(884.12) = -3.11, p = 0.002 |
| Fat (g) | 3.19 ± 5.91 | 439 | 2.75 ± 4.77 | 571 | t(827.93) = -1.28, p = 0.201 |
| *Saturated fat (g)* | 0.40 ± 1.51 | 366 | 0.70 ± 1.99 | 404 | t(745.54) = 2.42, p = 0.016 |
| *Monounsaturated fat (g)* | - | 0 | 2.22 ± 1.86 | 3 | - |
| *Polyunsaturated fat (g)* | 0.47 | 1 | 0.78 ± 0.39 | 5 | - |
| *Omega-3 (g)* | 0.23 | 2 | 0.09 | 1 | - |
| *Omega-6 (g)* | - | 0 | 0.35 | 1 | - |
| *Trans fat (g)* | 0.00 ± 0.01 | 368 | 0.00 ± 0.01 | 362 | t(582.55) = -0.56, p = 0.576 |
| *Cholesterol (mg)* | 29.60 ± 48.09 | 9 | 10.43 ± 9.77 | 75 | t(8.08) = -1.19, p = 0.267 |
| Carbohydrates (g) | 35.06 ± 29.89 | 439 | 28.62 ± 28.22 | 571 | t(914.22) = -3.48, p<0.001 |
| *Sugar (g)* | 13.19 ± 13.22 | 437 | 10.56 ± 11.65 | 571 | t(872.53) = -3.29, p = 0.001 |
| *Added sugar (g)* | 12.15 ± 13.73 | 41 | 6.49 ± 9.99 | 11 | t(21.33) = -1.53, p = 0.141 |
| Fiber (g) | 2.57 ± 3.47 | 430 | 2.85 ± 3.69 | 538 | t(941.37) = 1.23, p = 0.220 |
| Protein (g) | 3.56 ± 4.97 | 439 | 4.11 ± 4.64 | 571 | t(908.51) = 1.81, p = 0.071 |
| Calcium (mg) | 70.53 ± 144.43 | 411 | 56.80 ± 115.48 | 519 | t(773.35) = -1.57, p = 0.117 |
| Calcium (%) | 22.49 ± 52.05 | 411 | 15.50 ± 38.07 | 519 | t(727.64) = -2.28, p = 0.023 |
| Magnesium (mg) | 73.49 ± 107.49 | 63 | 59.91 ± 76.68 | 12 | t(20.27) = -0.52, p = 0.607 |
| Magnesium (%) | 97.04 ± 143.20 | 63 | 80.03 ± 102.66 | 12 | t(20.17) = -0.49, p = 0.629 |
| Phosphorus (mg) | 261.20 ± 161.16 | 15 | 376.01 ± 152.60 | 10 | t(20.19) = 1.80, p = 0.087 |
| Phosphorus (%) | 90.76 ± 63.96 | 15 | 135.07 ± 57.88 | 10 | t(20.77) = 1.80, p = 0.087 |
| Potassium (mg) | 234.72 ± 205.22 | 413 | 275.74 ± 219.96 | 502 | t(890.90) = 0.78, p = 0.436 |
| Potassium (%) | 24.88 ± 25.31 | 413 | 24.88 ± 25.16 | 502 | t(877.39) = -0.00, p = 0.999 |
| Sodium (mg) | 50.26 ± 91.60 | 439 | 37.69 ± 66.02 | 571 | t(764.12) = -2.43, p = 0.015 |
| Choline (mg) | 159.03 ± 170.70 | 40 | 471.43 | 1 | - |
| Choline (%) | 97.98 ± 106.36 | 40 | 285.71 | 1 | - |
| Copper (mg) | 0.23 ± 0.25 | 23 | 0.07 ± 0.04 | 4 | t(24.99) = -2.90, p = 0.008 |
| Copper (%) | 107.43 ± 117.06 | 23 | 33.33 ± 19.44 | 4 | t(24.93) = -2.82, p = 0.009 |
| Iron (mg) | 5.14 ± 10.58 | 418 | 2.75 ± 8.22 | 516 | t(774.60) = -3.78, p<0.001 |
| Iron (%) | 51.31 ± 102.95 | 418 | 28.69 ± 87.17 | 516 | t(818.46) = -3.57, p<0.001 |
| Iodide (ug) | 25.76 ± 39.38 | 49 | 25.48 ± 40.19 | 7 | t(7.74) = -0.02, p = 0.987 |
| Iodide (%) | 22.24 ± 31.89 | 49 | 19.60 ± 30.92 | 7 | t(7.94) = -0.21, p = 0.839 |
| Manganese (mg) | 0.13 ± 0.30 | 25 | 4.67 ± 5.57 | 8 | t(7.01) = 2.30, p = 0.055 |
| Manganese (%) | 21.90 ± 50.55 | 25 | 59.03 ± 45.84 | 8 | t(12.94) = 1.94, p = 0.074 |
| Selenium (mcg) | 10.66 ± 13.60 | 22 | 11.22 ± 6.14 | 4 | t(9.65) = 0.13, p = 0.896 |
| Selenium (%) | 48.26 ± 64.81 | 22 | 56.10 ± 30.67 | 4 | t(9.00) = 0.38, p = 0.713 |
| Zinc (mg) | 3.08 ± 3.38 | 90 | 1.80 ± 1.77 | 23 | t(67.58) = -2.51, p = 0.015 |
| Zinc (%) | 102.02 ± 108.67 | 90 | 60.42 ± 59.91 | 23 | t(63.45) = -2.45, p = 0.017 |
| Vitamin A (ug) | 196.49 ± 181.31 | 90 | 198.53 ± 404.21 | 91 | t(125.11) = 0.04, p = 0.965 |
| Vitamin A (%) | 43.22 ± 41.28 | 90 | 42.70 ± 78.96 | 91 | t(136.10) = -0.06, p = 0.956 |
| Vitamin B1 (mg) | 0.92 ± 0.61 | 66 | 1.31 ± 1.18 | 39 | t(50.34) = 1.90, p = 0.063 |
| Vitamin B1 (%) | 282.74 ± 195.51 | 66 | 360.40 ± 264.63 | 39 | t(62.66) = 1.59, p = 0.116 |
| Vitamin B2 (mg) | 1.25 ± 0.76 | 60 | 1.32 ± 0.81 | 17 | t(24.64) = 0.29, p = 0.776 |
| Vitamin B2 (%) | 304.66 ± 186.84 | 60 | 315.87 ± 189.92 | 17 | t(25.46) = 0.22, p = 0.831 |
| Vitamin B3 (mg) | 9.25 ± 7.89 | 87 | 14.73 ± 13.72 | 25 | t(28.71) = 1.91, p = 0.066 |
| Vitamin B3 (%) | 219.32 ± 193.46 | 87 | 289.43 ± 223.33 | 25 | t(35.01) = 1.42, p = 0.163 |
| Vitamin B5 (mg) | 3.46 ± 2.40 | 12 | 1.50 ± 0.90 | 9 | t(14.83) = -2.59, p = 0.020 |
| Vitamin B5 (%) | 178.97 ± 121.47 | 12 | 81.48 ± 45.73 | 9 | t(14.82) = -2.55, p = 0.022 |
| Vitamin B6 (mg) | 0.45 ± 1.08 | 61 | 0.20 ± 0.18 | 19 | t(69.31) = -1.69, p = 0.096 |
| Vitamin B6 (%) | 94.56 ± 63.16 | 61 | 56.08 ± 45.62 | 19 | t(41.47) = -2.91, p = 0.006 |
| Vitamin B7 (ug) | 5.04 ± 3.38 | 34 | 8.73 ± 10.60 | 11 | t(10.67) = 1.14, p = 0.281 |
| Vitamin B7 (%) | 80.67 ± 56.68 | 34 | 134.74 ± 176.73 | 11 | t(10.67) = 1.00, p = 0.340 |
| Vitamin B9 (ug) | 55.99 ± 72.20 | 52 | 14.44 ± 21.57 | 15 | t(64.83) = -3.63, p = 0.001 |
| Vitamin B9 (%) | 79.57 ± 89.43 | 52 | 38.00 ± 43.91 | 15 | t(48.49) = -2.47, p = 0.017 |
| Vitamin B12 (ug) | 2.34 ± 15.89 | 70 | 0.58 ± 0.20 | 4 | t(69.35) = -0.93, p = 0.358 |
| Vitamin B12 (%) | 79.16 ± 86.07 | 70 | 77.38 ± 59.53 | 4 | t(3.76) = -0.06, p = 0.958 |
| Vitamin C (mg) | 22.83 ± 20.29 | 118 | 22.65 ± 23.67 | 80 | t(151.88) = -0.06, p = 0.954 |
| Vitamin C (%) | 54.06 ± 42.09 | 118 | 55.94 ± 53.41 | 80 | t(142.48) = 0.26, p = 0.792 |
| Vitamin D (ug) | 4.17 ± 4.66 | 45 | 2.78 ± 3.04 | 56 | t(72.45) = -1.71, p = 0.091 |
| Vitamin D (%) | 40.77 ± 43.67 | 45 | 21.71 ± 32.16 | 56 | t(78.74) = -2.44, p = 0.017 |
| Vitamin E (mg) | 4.19 ± 4.09 | 88 | 4.90 ± 2.48 | 17 | t(35.48) = 0.95, p = 0.348 |
| Vitamin E (%) | 77.97 ± 76.03 | 88 | 85.66 ± 50.57 | 17 | t(31.91) = 0.52, p = 0.605 |
| Vitamin K (ug) | 2.92 ± 1.21 | 9 | 12.99 ± 15.24 | 14 | t(13.26) = 2.46, p = 0.028 |
| Vitamin K (%) | 101.48 ± 56.54 | 9 | 283.45 ± 490.96 | 14 | t(13.53) = 1.37, p = 0.192 |

¹Values are presented as mean ± standard deviation.

**Supplementary Table 6. Comparative Analysis of Gluten-free and Gluten-containing Products**

|  | Gluten-free¹ | n | Gluten-containing | n | T-test |
| --- | --- | --- | --- | --- | --- |
| Serving size (g or mL) | 100.00 | 800 | 100.00 | 210 | - |
| Calories (kcal) | 140.43 ± 143.06 | 800 | 265.29 ± 171.49 | 210 | t(289.80) = -9.70, p < 0.001 |
| Fat (g) | 2.16 ± 4.76 | 800 | 5.92 ± 6.13 | 210 | t(278.36) = -8.26, p < 0.001 |
| *Saturated fat (g)* | 0.43 ± 1.66 | 642 | 1.18 ± 2.21 | 128 | t(156.94) = -3.61, p < 0.001 |
| *Monounsaturated fat (g)* | 2.22 ± 1.86 | 3 | - | - | - |
| *Polyunsaturated fat (g)* | 0.72 ± 0.37 | 6 | - | - | - |
| *Omega-3 (g)* | 0.19 ± 0.08 | 3 | - | - | - |
| *Omega-6 (g)* | 0.35 | 1 | - | - | - |
| *Trans fat (g)* | 0.00 ± 0.01 | 620 | 0.00 ± 0.02 | 110 | t(115.19) = -1.63, p = 0.106 |
| *Cholesterol (mg)* | 13.06 ± 20.80 | 64 | 10.64 ± 7.90 | 20 | t(78.88) = 0.77, p = 0.443 |
| Carbohydrates (g) | 27.66 ± 28.00 | 800 | 45.77 ± 28.89 | 210 | t(319.74) = -8.14, p < 0.001 |
| *Sugar (g)* | 11.35 ± 13.00 | 798 | 13.02 ± 9.81 | 210 | t(423.33) = -2.03, p = 0.042 |
| *Added sugar (g)* | 10.73 ± 14.61 | 33 | 11.32 ± 10.52 | 19 | t(47.34) = -0.17, p = 0.867 |
| Fiber (g) | 2.54 ± 3.62 | 768 | 3.45 ± 3.44 | 200 | t(323.15) = -3.32, p < 0.001 |
| Protein (g) | 3.11 ± 4.34 | 800 | 6.76 ± 5.34 | 210 | t(285.49) = -9.13, p < 0.001 |
| Calcium (mg) | 52.98 ± 122.21 | 741 | 101.64 ± 147.61 | 119 | t(257.48) = -4.18, p < 0.001 |
| Calcium (%) | 14.76 ± 41.33 | 741 | 33.60 ± 54.36 | 119 | t(246.16) = -4.45, p < 0.001 |
| Magnesium (mg) | 42.06 ± 53.90 | 42 | 108.55 ± 134.86 | 33 | t(40.05) = -2.67, p = 0.011 |
| Magnesium (%) | 55.18 ± 70.24 | 42 | 144.12 ± 180.43 | 33 | t(39.64) = -2.68, p = 0.011 |
| Phosphorus (mg) | 297.47 ± 190.45 | 11 | 314.71 ± 148.78 | 14 | t(18.60) = -0.25, p = 0.808 |
| Phosphorus (%) | 107.30 ± 74.39 | 11 | 109.41 ± 58.08 | 14 | t(18.59) = -0.08, p = 0.939 |
| Potassium (mg) | 240.99 ± 227.27 | 734 | 239.99 ± 142.39 | 176 | t(416.95) = 0.07, p = 0.942 |
| Potassium (%) | 24.50 ± 26.18 | 734 | 26.51 ± 20.70 | 176 | t(322.63) = -1.10, p = 0.273 |
| Sodium (mg) | 32.34 ± 67.43 | 800 | 84.35 ± 100.62 | 210 | t(260.24) = -7.08, p < 0.001 |
| Choline (mg) | 142.57 ± 168.06 | 35 | 307.14 ± 162.82 | 6 | t(6.96) = -2.28, p = 0.057 |
| Choline (%) | 85.72 ± 101.45 | 35 | 200.79 ± 107.51 | 6 | t(6.62) = -2.44, p = 0.047 |
| Copper (mg) | 0.22 ± 0.25 | 24 | 0.08 ± 0.04 | 3 | t(22.27) = 2.58, p = 0.017 |
| Copper (%) | 103.79 ± 115.87 | 24 | 37.78 ± 21.17 | 3 | t(20.29) = 2.48, p = 0.022 |
| Iron (mg) | 1.83 ± 6.12 | 743 | 11.55 ± 14.64 | 191 | t(207.36) = -8.98, p < 0.001 |
| Iron (%) | 19.16 ± 66.51 | 743 | 115.23 ± 140.81 | 191 | t(212.24) = -9.17, p < 0.001 |
| Iodide (ug) | 39.78 ± 52.76 | 24 | 15.17 ± 19.50 | 32 | t(27.74) = 2.18, p = 0.038 |
| Iodide (%) | 34.24 ± 42.46 | 24 | 12.66 ± 15.15 | 32 | t(27.46) = 2.39, p = 0.024 |
| Manganese (mg) | 0.64 ± 2.46 | 29 | 5.53 ± 5.48 | 4 | t(3.17) = -1.76, p = 0.172 |
| Manganese (%) | 26.27 ± 48.46 | 29 | 64.48 ± 67.09 | 4 | t(3.45) = -1.10, p = 0.342 |
| Selenium (mcg) | 10.74 ± 12.64 | 26 | - | 0 | - |
| Selenium (%) | 49.47 ± 60.41 | 26 | - | 0 | - |
| Zinc (mg) | 2.53 ± 2.93 | 47 | 3.03 ± 3.31 | 66 | t(105.74) = -0.84, p = 0.401 |
| Zinc (%) | 86.27 ± 94.14 | 47 | 98.74 ± 107.40 | 66 | t(106.21) = -0.65, p = 0.514 |
| Vitamin A (ug) | 211.22 ± 334.66 | 145 | 142.31 ± 197.56 | 36 | t(91.36) = 1.60, p = 0.113 |
| Vitamin A (%) | 46.00 ± 27.21 | 145 | 30.72 ± 39.81 | 36 | t(90.99) = 1.76, p = 0.081 |
| Vitamin B1 (mg) | 1.06 ± 1.41 | 27 | 1.07 ± 0.62 | 78 | t(29.57) = -0.02, p = 0.986 |
| Vitamin B1 (%) | 262.05 ± 274.05 | 27 | 328.73 ± 205.49 | 78 | t(36.64) = -1.16, p = 0.255 |
| Vitamin B2 (mg) | 0.74 ± 0.46 | 18 | 1.43 ± 0.77 | 59 | t(48.39) = -4.71, p < 0.001 |
| Vitamin B2 (%) | 180.81 ± 117.91 | 18 | 345.67 ± 186.95 | 59 | t(45.27) = -4.46, p < 0.001 |
| Vitamin B3 (mg) | 7.54 ± 11.74 | 44 | 12.37 ± 7.64 | 68 | t(66.62) = -2.42, p = 0.018 |
| Vitamin B3 (%) | 149.12 ± 196.37 | 44 | 290.52 ± 186.04 | 68 | t(88.33) = -3.80, p = 0.0003 |
| Vitamin B5 (mg) | 4.58 ± 1.69 | 7 | 1.64 ± 1.57 | 14 | t(11.29) = 3.83, p = 0.003 |
| Vitamin B5 (%) | 239.45 ± 80.09 | 7 | 86.05 ± 78.25 | 14 | t(11.85) = 4.17, p = 0.001 |
| Vitamin B6 (mg) | 0.26 ± 0.20 | 51 | 0.62 ± 1.55 | 29 | t(28.54) = -1.25, p = 0.222 |
| Vitamin B6 (%) | 73.96 ± 51.73 | 51 | 105.59 ± 72.21 | 29 | t(44.60) = -2.08, p = 0.044 |
| Vitamin B7 (ug) | 6.10 ± 5.07 | 11 | 5.89 ± 6.41 | 34 | t(21.27) = 0.11, p = 0.914 |
| Vitamin B7 (%) | 92.85 ± 82.27 | 11 | 94.22 ± 106.59 | 34 | t(21.86) = -0.05, p = 0.965 |
| Vitamin B9 (ug) | 51.31 ± 77.96 | 41 | 39.41 ± 43.42 | 26 | t(64.15) = 0.80, p = 0.426 |
| Vitamin B9 (%) | 71.30 ± 99.22 | 41 | 68.62 ± 49.43 | 26 | t(62.19) = 0.15, p = 0.884 |
| Vitamin B12 (ug) | 0.57 ± 0.56 | 33 | 3.59 ± 20.77 | 41 | t(40.07) = -0.93, p = 0.357 |
| Vitamin B12 (%) | 94.82 ± 97.86 | 33 | 66.38 ± 70.80 | 41 | t(56.60) = 1.40, p = 0.167 |
| Vitamin C (mg) | 23.11 ± 20.45 | 150 | 21.67 ± 25.27 | 48 | t(67.83) = 0.36, p = 0.721 |
| Vitamin C (%) | 55.83 ± 46.02 | 150 | 51.69 ± 49.82 | 48 | t(74.42) = 0.51, p = 0.612 |
| Vitamin D (ug) | 2.85 ± 3.50 | 89 | 7.48 ± 4.38 | 12 | t(12.96) = -3.52, p = 0.004 |
| Vitamin D (%) | 24.19 ± 33.98 | 89 | 74.83 ± 43.81 | 12 | t(12.85) = -3.85, p = 0.002 |
| Vitamin E (mg) | 5.15 ± 4.24 | 49 | 3.57 ± 3.38 | 56 | t(91.45) = 2.09, p = 0.040 |
| Vitamin E (%) | 97.68 ± 79.88 | 49 | 63.06 ± 61.36 | 56 | t(89.53) = 2.46, p = 0.016 |
| Vitamin K (ug) | 6.63 ± 10.22 | 20 | 25.18 ± 18.77 | 3 | t(2.18) = -1.67, p = 0.226 |
| Vitamin K (%) | 93.00 ± 41.79 | 20 | 1,077.20 ± 750.70 | 3 | t(2.00) = -2.11, p = 0.169 |

¹Values are presented as mean ± standard deviation.
